# Effects of *Salmonella Typhimurium* infection on intestinal flora and intestinal tissue arachidonic acid metabolism in Wenchang chickens

**DOI:** 10.3389/fmicb.2025.1514115

**Published:** 2025-01-24

**Authors:** Shenghong Chen, Yaochen Xie, Dingqian Guo, Tiansen Li, Zhen Tan, Xuhua Ran, Xiaobo Wen

**Affiliations:** Hainan Key Lab of Tropical Animal Reproduction and Breeding and Epidemic Disease Research, Department of Animal Science and Technology, School of Tropical Agriculture and Forestry (School of Agricultural and Rural Affairs, School of Rural Revitalization), Hainan University, Haikou, China

**Keywords:** *Salmonella Typhimurium*, gut microbiota, lipid oxidation, arachidonic acid, Wenchang chicken

## Abstract

*Salmonella* infections can lead to intestinal inflammation and metabolic disorders in birds. However, whether arachidonic acid (ARA) metabolism is involved in *Salmonella*-induced intestinal inflammation remains unclear. This experiment investigated the changes in cecal flora and ARA metabolism in Hainan Wenchang chickens infected with *S. Typhimurium* using 16s rDNA sequencing and targeted metabolomics. The results showed that the levels of ARA metabolites were increased in the cecum tissue of Wenchang chickens after infection with *S. Typhimurium*, including prostaglandin E2 (PGE_2_), prostaglandin F2α (PGF_2α_), lipoxin A4 (LXA4), ± 8(9)-EET, ± 11(12)-EET, and ± 8,9-DiHETrE. The content of key enzymes for ARA production and metabolism (Phospholipase A2 PLA2 and Cyclooxygenase-2 COX-2) in chicken cecum tissues was increased after *S. Typhimurium* infection. The relative mRNA levels of inflammatory factors were also increased after infection, including Interferon-γ (IFN-γ), Transforming growth factor-β1 (TGF-β1), Interleukin-4 (IL-4), and Interleukin-6 (IL-6). In HD11 cells, the use of a cyclooxygenase (COX) inhibitor reduced the increased levels of COX-2 and PGF_2α_ induced by *S. Typhimurium* infection and effectively reduced the inflammatory response. In addition, the number of beneficial genera (e.g., *Bifidobacterium*, *Lactobacillus*, and *Odorobacterium*) in the cecum of Wenchang chickens was significantly reduced after infection with *S. Typhimurium*. The present study revealed the structure of cecal flora in *S. Typhimurium*-infected Wenchang chickens. In addition, this study demonstrated that *S. Typhimurium* activates the ARA cyclooxygenase metabolic pathway, which in turn mediates the development of intestinal inflammation in Wenchang chickens. The results can provide data support and theoretical support for the prevention and control of avian salmonellosis.

## 1 Introduction

*Salmonella* is one of the most common intestinal pathogens in animals and humans. In poultry, *Salmonella enteritidis* (*S. Enteritidis*) and *Salmonella Typhimurium* (*S. Typhimurium*) infections are insidious and usually asymptomatic or cause intestinal inflammation (Shaji et al., 2023). Moreover, *Salmonella spp.* can compete with the normal microbiota in various ways, disrupting its balance and causing a decline in the immunity of livestock and poultry, which, in severe cases, can cause high mortality (Winter et al., 2010; Fàbrega and Vila, 2013). Salmonellosis reduces the body weight of broilers and the egg production of laying hens, as well as reduces the fertility of chickens and the hatchability of chicks, all of which can have a significant impact on the poultry industry and cause economic losses (El-Saadony et al., 2022).

The ability of *Campylobacter* to infect chickens is influenced by gut flora (Han et al., 2017), suggesting that gut microbial communities play an important role in avian health. Available studies have shown that expression of the pro-inflammatory cytokine interleukin 6 (IL-6) is positively correlated with *Shigella*, *Parasuttterella*, and *Vampirovibrio* and negatively correlated with *Fecalibacterium* (Oakley and Kogut, 2016), which shows that the immune response of the host is strongly correlated with changes in the abundance of intestinal bacteria. Therefore, it is essential to have a comprehensive understanding of the effects of *Salmonella* infection on the intestinal flora of poultry, which can help control the disease’s occurrence and development. Wenchang chickens are the most economically valuable indigenous livestock species in Hainan Province (Gu et al., 2022). However, little is known about the changes in their intestinal flora after *Salmonella* infection.

The ability of *S. Typhimurium* to use the respiratory electron acceptor tetrathionate, which is produced during inflammation, provides it with a growth advantage over other flora in the inflamed gut environment (Winter et al., 2010). Thus, controlling the development of intestinal inflammation might help enhance resistance to intestinal colonization by *Salmonella* (Winter et al., 2010). The arachidonic acid (ARA) metabolic pathway is major pathway for the production of inflammatory mediators (Monk et al., 2012; Monk et al., 2014). And inflammatory mediators are involved in the onset and development of inflammatory responses. ARA and its metabolic derivatives (ARA-like) are involved in physiological processes such as blood leukocyte chemotaxis, platelet aggregation, and the promotion of intestinal inflammation by mediating Th17 and Th1 cells (Monk et al., 2014; Isse et al., 2022). This suggests that ARA-like is nvolved in pathophysiological processes related to cell injury, inflammation, and apoptosis. Infection of human intestinal epithelial cell lines (Caco2 and HT29) by entero-invasive *Escherichia coli* and *Salmonella* significantly increased cyclooxygenase-2 expression and prostaglandin E2 (PGE_2_) production (Resta-Lenert and Barrett, 2002). Increased prostaglandin F2α (PGF_2α_) release was also observed in intestinal epithelial cells following *Salmonella* infection (Eckmann et al., 1997). PGE_2_, 5-HETE, 8-HETE, 12-HETE, and 15-HETE syntheses were increased in Caco2 cells after lipopolysaccharide (10 μg/mL) treatment (Le Faouder et al., 2013). However, the question of whether ARA metabolites mediate enteritis caused by *Salmonella* infection in poultry has not been reported. Therefore, given the critical role of ARA and its metabolites in mediating the inflammatory response, it is essential to elucidate the metabolic changes of ARA in intestinal tissues following *Salmonella* infection. The metabolic changes of ARA in the intestinal tissues of Wenchang chickens after *S. Typhimurium* infection were detected by ultra-high performance liquid chromatography-tandem mass spectrometry (UHPLC-MS/MS). This study further explored the potential link between ARA metabolic pathways and enteritis caused by *Salmonella* infections in poultry, which might contribute to the prevention and treatment of the disease.

## 2 Materials and methods

### 2.1 Ethics statement

The sampling method and all subsequent methods were approved by the Ethics Committee of Hainan University (Haikou, China, Permit No. HNUAUCC-2023-00080). This experiment did not involve endangered or protected species.

### 2.2 Animal experiments

**FIGURE 1:**
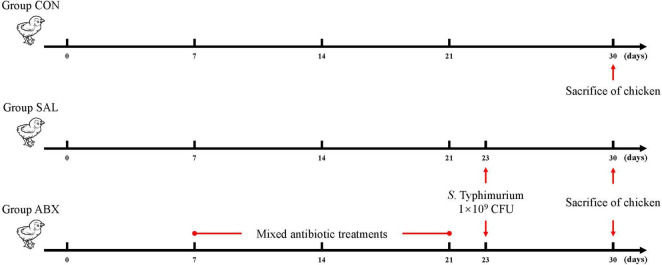
Schematic diagram of chicken grouping and experimental procedure.

A total of 36 14 day old Wenchang chickens (purchased from Hainan Taniu Wenchang Chicken Co.) were randomly divided into three groups (*n* = 12). The experiment started on day 0 (14 days of age) and ended on day 30 (44 days of age), with a duration of 30 days. Grouping programs: The CON group was the healthy control group. The SAL group was infected with *Salmonella Typhimurium* (*S. Typhimurium*) on day 23. The ABX group was administered a cocktail of antibiotics containing ampicillin (1 g/L), neomycin sulfate (1 g/L), metronidazole (1 g/L), and vancomycin (0.5 g/L) via drinking water from days 7 to 21; on day 22, this group was given regular drinking water and infected with *S. Typhimurium* on day 23 (Figure 1). Infection was by gavage and the infection measure was 1 × 10^9^ CFU of *S. Typhimurium* per chicken. The strain was *Salmonella enterica serovar Typhimurium* (strain no. ATCC14028). Chicken sera were collected from all groups on the 24th day after the start of the experiment and checked for successful infection using a Chicken Salmonella ELISA KIT (Shanghai Yaji Biotechnology Co., Ltd., shanghai, China). If the OD450 reading was greater than the Cut-off value of 0.2, then the chicken was infected with *S. Typhimurium* (Supplementary Figure S1). The three groups of chickens were kept in three separate rooms with the same temperature, humidity, and other conditions to avoid cross-infection. Room information: At 3–4 weeks of age, the temperature was 28–30°C, the relative humidity was 55–60%, and the illumination time was 16 h; at 5–6 weeks of age, the temperature was 24–26°C, the relative humidity was 50–55%, and the light time was 12 h. Chickens were fed in cages with dimensions of 120 cm × 60 cm × 45 cm. Feeding management: all the chickens were fed the same diet and had free access to food and water. On day 30, 6 chickens per group were randomly selected for cervical dislocation euthanasia, and cecum contents and cecum intestinal tissues were aseptically collected on ice for 16s rDNA sequencing analysis and targeted metabolomics analysis.

### 2.3 16s rDNA sequencing and data analysis

The DNA was extracted using the CTAB (hexadecyl trimethyl ammonium bromide) method according to the manufacturer’s instructions. PCR amplification was used to construct libraries for sequencing. Briefly, amplification was performed using the specific primers listed in Table 1, and the PCR products were confirmed using 2% agarose gel electrophoresis, purified with AMPure XT beads (Beckman Coulter Genomics, Danvers, MA, United States), and quantified with Qubit (Invitrogen, United States) to obtain libraries for sequencing. The size and number of libraries were evaluated using Agilent 2100 Bioanalyzer (Agilent, United States) and Illumina (Kapa Biosciences, Woburn, MA, United States) library quantification kits, respectively. The libraries were sequenced on the NovaSeq PE250 platform. Quality raw reads were filtered under specific filtering conditions to obtain high-quality clean tags according to fqtrim (v0.94). Chimeric sequences were filtered using Vsearch software (v2.3.4) (Rognes et al., 2016). After dereplication using DADA2 (Callahan et al., 2016), we obtained the feature table and sequence. Alpha and beta diversities were randomly calculated by normalizing to identical sequences. Then, according to the SILVA (Release 138) classifier (Pruesse et al., 2012), feature abundance was normalized using the relative abundance of each sample. QIIME2 was used to calculate beta diversity and alpha diversity (Chao1, observed species, Good’s coverage index, Shannon, and Simpson) (Bolyen et al., 2019), and the R package was used to prepare the graphs. BLAST was used for sequence alignment, and the feature sequences were annotated using the SILVA database for each representative sequence. Other diagrams were drawn using the R package (v3.5.2).^1^ This study’s raw data were uploaded to the NCBI database under the number PRJNA1027078. Sequencing and data analysis were entrusted to Shanghai Biotree Biotech Co.

**TABLE 1:** PCR-specific amplification primer sequences.

Amplified fragments	Primer sequences
V3–V4	F (5′-CCTACGGGNGGCWGCAG-3′)
	R (5′-GACTACHVGGGTATCTAATCC-3′)
V4	F (5′-GTGYCAGCMGCCGCGGTAA-3′)
	R (5′-GGACTACHVGGGTWTCTAAT-3′)
V4–V5	F (5′-GTGCCAGCMGCCGCGG-3′)
	R (5′-CCGTCAATTCMTTTRAGTTT-3′)
Archae	F (5′-GYGCASCAGKCGMGAAW-3′)
	R (5′-GGACTACHVGGGTWTCTAAT-3′)

LDA effect size (LEfSe) analysis was used to find species that differed significantly in abundance between groups. First, the Kruskal-Wallis rank sum test was utilized to detect all characterized species, and significantly different species were identified by detecting differences in species abundance between different groups. Then, the Wilcoxon rank sum test was utilized to test whether all subspecies of the significantly different species obtained in the previous step converged to the same taxonomic level. Finally, linear discriminant analysis (LDA) was used to estimate the magnitude of the effect of species abundance on the differential effect to obtain the final differential species. The screening conditions for LDA were LDA > 4 and *P* < 0.05.

### 2.4 Metabolite extraction and standard solution preparation

Briefly, the intestinal tissues were treated with extract (80% methanol/H_2_O (V/V), precooled at −40°C, containing isotopically labeled internal standard mixture), purified via solid-phase extraction, dried through nitrogen blowing, re-solubilized with 30% acetonitrile, and then centrifuged at 4°C and 12,000 rpm for 15 min to obtain the supernatant, which was subjected to UHPLC-MS/MS analysis.

Stock solutions were prepared by dissolving or diluting each standard substance to a final concentration of 1 μg/mL. An aliquot of each stock solution was transferred to an Eppendorf tube to obtain a mixed working standard solution. A series of traditional calibration solutions were then prepared through stepwise dilution of this mixed standard solution (containing the isotopically labeled internal standard mixture at identical concentrations to the samples). Supplementary Table S2 lists the standards used in this assay.

### 2.5 UHPLC-MRM-MS analysis

UHPLC separation was performed using an EXIONLC System (Sciex) equipped with a Waters ACQUITY UPLC BEH C18 column (150 × 2.1 mm, 1.7 μm, Waters). A SCIEX 6500 QTRAP+ triple quadrupole mass spectrometer (Sciex) equipped with an IonDrive Turbo V electrospray ionization interface was used for assay development. The MRM parameters for each target analyte were optimized using flow injection analysis by injecting standard solutions of individual analytes into the API source of the mass spectrometer. Several of the most sensitive transitions were used in MRM scan mode to optimize the collision energy for each Q1/Q3 pair (Supplementary Table S3). Among the optimized MRM transitions per analyte, the Q1/Q3 pairs with the highest sensitivity and selectivity were selected as “quantifiers” for quantitative monitoring. The additional transitions acted as qualifiers to verify the identity of the target analyte. The SCIEX Analyst WorkStation Software (Version 1.6.3) and the Multiquant 3.03 software were used for MRM data acquisition and processing. Metabolite assays and data analysis were entrusted to Shanghai Biotree Biotech Co.

### 2.6 Metabolite assay results and quality control

The calibration solutions were sequentially diluted 2-fold and analyzed using UHPLC-MRM-MS. The lower limits of detection (LLODs) and quantification (LLOQs) of the methods were calculated using their signal-to-noise ratios. According to the United States Food and Drug Administration guidelines for bioanalytical method validation, the LLODs of a method are defined as the concentration of a compound with a signal-to-noise ratio of 3, and the LLOQs of a method are defined as the concentration of a compound with a signal-to-noise ratio of 10. Supplementary Table S4 lists the resulting LLODs and LLOQs. The LLODs for the targeted metabolites ranged from 0.0195 to 1.2500 ng/mL, and the LLOQs ranged from 0.0390 to 2.5000 ng/mL. The correlation coefficients (R2) of regression fitting were above 0.9959 for the analytes, indicating a good quantitative relationship between the MS responses and analyte concentrations, satisfying the target metabolomics analysis. Supplementary Table S5 lists the analytical recoveries and relative standard deviations of the QC samples with five technical replicates. The recoveries were 85.07–111.47% for all analytes, with all RSDs below 14.09% (*n* = 5). The analysis metrics indicated that the method allowed for the accurate quantitation of the targeted metabolites in the biological samples in the above concentration range.

The quantification results are presented in Supplementary Table S6. The final concentration (cF, ng/mL) was equal to the calculated concentration (cC, ng/mL) multiplied by the dilution factor (Dil). For solid samples, the metabolite concentration (cM, ng/g) was equal to the final concentration (cF, ng/mL) multiplied by the final volume (VF, μL) and equal to the sample experiment concentration factor (CF) divided by the mass (m, mg) of the sample. N/A indicates that the targeted metabolites were not detectable in the corresponding samples.

### 2.7 Cell experiments

#### 2.7.1 Establishment of HD11 cell model infected by *S. Typhimurium*

First, the lowest concentration of cyclooxygenase inhibitor (aspirin) that inhibited the growth of *S. Typhimurium* in vitro was determined. *S. Typhimurium* was cultured in an LB (Luria-Bertani) sterile liquid medium containing different aspirin concentrations for 6–8 h, and the bacteria growth in each group was counted by the plate count method.

Varied concentrations of aspirin (0, 31.25, 62.5, 125, 200, 500, and 1000 μg/mL) were then applied to HD11 cells for different times (0, 12, and 24 h). Cell viability was measured by MTT to determine the maximal aspirin concentration and treatment time.

Finally, HD11 cells were infected with *S. Typhimurium* at selected MOIs (1, 5, 10, 20, and 50). The relative expression levels of iNOS, IL-1β, IL-6, and IL-10 mRNA in the cells were measured to determine the appropriate MOIs and time of infection.

#### 2.7.2 Chicken grouping and cell experiments

HD11 cells were infected with *S. Typhimurium* for 2 h (MOI = 20) after being pretreated with a medium with aspirin concentrations of 30, 60, and 120 μg/mL for 12 h.

### 2.8 Quantitative real-time polymerase chain reactionanalysis and ELISA assay

Changes in specific cytokine mRNAs and ARA metabolites in cecum in vivo and HD11 cells in vitro following *S. Typhimurium* infection were assessed by qRT-PCR and ELISA. The cecum tissue was homogenized using an automated sample rapid grinder (Tissuelyser-24L, Shanghai Jingxin Industrial Development Co., Ltd., Shanghai, China), and total RNA was extracted from the cecum tissue using the TRIzol method. Total RNA was extracted from HD11 cells by the TRIzol method. cDNA was prepared by the TIANGEN cDNA synthesis kit. The qRT-PCR analysis was performed using SYBR Green Master Mix, and glyceraldehyde-3-phosphate dehydrogenase (GAPDH) was used as a housekeeping gene. The mRNA concentration of all samples was adjusted to 1,000 ng/mL during reverse transcription. The primers used are listed in Supplementary Table S1. The relative gene expression was calculated using the 2^–ΔΔ*Ct*^ method. GraphPad Prism (version 8.0c) and SPSS 17.0 were used for statistical analysis. Data were analyzed using the 2^–ΔΔ*Ct*^ method and expressed as mean ± standard error (SEM). The statistical significance of the data was assessed using a one-way analysis of variance (ANOVA) followed by Duncan’s and Tukey’s tests.

The ELISA kits (Xiamen Lunchangshuo Biotech Co., Ltd., Xiamen, China) were used to detect COX-2, PGF2α, PLA2, and ARA in the samples according to the instructions.

### 2.9 Statistical analysis of data

Metabolites from each group were normalized using the Z-score normalization method, which is indicated by the value of (x-μ)/σ. “μ” denotes the mean of the overall data, “σ” denotes the standard deviation of the overall data, and “x” denotes the individual observation. The Z-score values were calculated using MedCalc 22^2^ software. R (3.4.4)^3^ was used to create a plot of the Z-scores.

Spearman’s correlation analysis was used to assess the correlation between three data types: oxidized fats, inflammatory factors, and intestinal flora. The Spearman correlation analysis was carried out using the Stats package (2.5.4)^4^ for R (3.4.4).

Multivariate statistical analysis was used to calculate the variable importance in the projection (VIP) values and orthogonal projections to latent structure discriminant analysis (OPLS-DA). The Mann–Whitney U test was used to compare differences between two groups of samples with biological replicates; the Kruskal–Wallis test was used to compare differences between multiple groups of samples with biological replicates. Adonis and ANOSIM were used to test whether the differences between groups were significantly greater than those within groups and to determine whether the subgroups were significant. The Vegan package (2.5.4)^5^ for R (3.4.4) was used for the above data analysis.

The datasets were evaluated for normal distribution using the D’Agostino–Pearson test, and presented as mean ± standard error of the mean (SEM). Statistical significance was determined with either a two-tailed unpaired Student’s *t*-test (for two groups) or one-way ANOVA (for grouped comparisons). In datasets analyzed using ANOVA, the Bonferroni post hoc test was adopted. A *p*-value less than 0.05 was considered statistically significant. All statistical analysis was performed with Prism version 8.0 (GraphPad, La Jolla, CA, United States) and SPSS 17.0.

## 3 Results

### 3.1 DNA sequence and microbial diversity index analysis

The number of Amplicon Sequence Variants (ASVs) was 4172, 3366, and 2242 in the CON, SAL, and ABX groups, respectively (Supplementary Figure S2A). The average number of ASVs in each group was 695, 561, and 374, respectively (*n* = 6). The Good’s coverage index values of all samples in the CON, SAL, and ABX groups exceeded 99% (Supplementary Figure S2B), which indicates that this sequencing result was representative of the real situation of the samples. When the number of valid sequences exceeded 40,000, the dilution curve (rarefaction curve) tended to flatten, indicating that the amount of sequencing data was saturated and that the depth and quantity of sequencing met the requirements for analysis (Supplementary Figure S2C). In addition, the rank abundance curves for each group of samples had a large span in the horizontal direction. They were relatively flat in the vertical direction, indicating good species richness and good homogeneity of the samples (Supplementary Figure S2D). The above results suggest that the quality of the sequencing data obtained in this study was reliable and sufficient, and the results are authentic.

**FIGURE 2:**
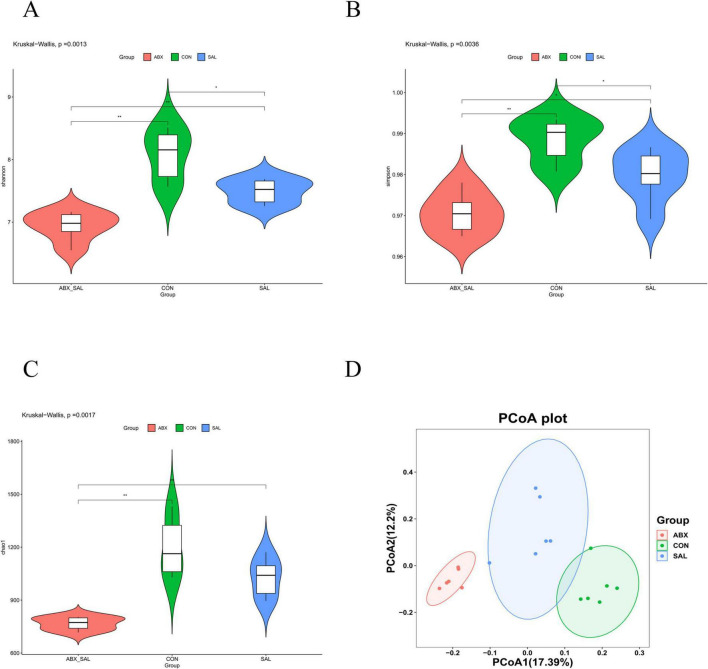
Results of difference analysis of cecum microbial communities between groups. **(A)** Shannon index. **(B)** Simpson’s index **(C)** Chao 1 index. **(D)** PCoA analysis results. Different colors represent different groups. The closer the samples are, the more similar the microbial composition is between the samples.

The results of the α-diversity analysis showed that the Shannon, Simpson, and Chao1 indices were lower in the SAL and ABX groups than in the CON group (Figures 2A–C). The principal coordinate analysis (PCoA) results among the three groups showed that the groups were distinct (*P* < 0.001) from each other and that there were differences in the structure of colony composition between the groups (Figure 2D).

### 3.2 Analysis of the composition of the intestinal microbial community

**FIGURE 3:**
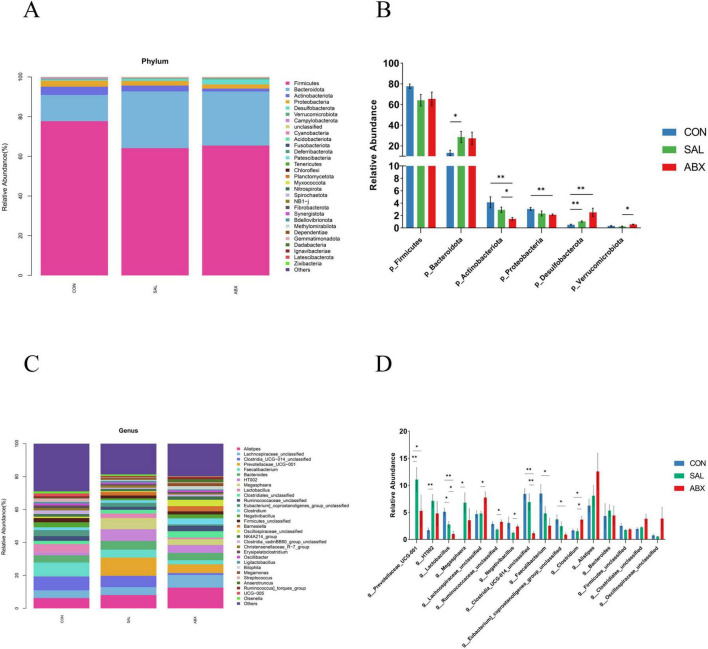
Taxonomic distribution of the CON, SAL, and ABX groups. **(A)** At the phylum level (top 30). **(B)** Differences in the partially dominant bacterial flora at the phylum level among the groups. **(C)** At the genus level (top 30). **(D)** Differences in the partially dominant bacterial flora at the genus level among the groups. “*” indicates a significant difference in statistics (*P < 0.05, **P < 0.01, and ***P < 0.001).

At the phylum level (Figures 3A,B), the relative abundance of *Cyanobacteria* (0.11%) and *Planctomycetota* (0.002%) decreased (*P* < 0.05), while the relative abundance of *Bacteroidota* (28.61%) and *Desulfobacterota* (1.04%) increased (*P* < 0.05) in the SAL group compared to the CON group. Relative abundance of *Proteobacteria* (2.12%), *Actinobacteria* (1.45%), and *Cyanobacteria* (0.06%) decreased (*P* < 0.05), while the relative abundance of *Desulfobacterota* (2.51%) increased (*P* < 0.05) in the ABX group compared to the CON group. For information on the abundance of the top 30 species at the level of phylum, order, family, and genus, refer to Supplementary Table S7. For information on differential species at the phylum and genus levels, refer to Supplementary Table S8.

At the genus level (Figures 3C,D), the relative abundance of 26 genera significantly increased and 40 genera significantly decreased in the SAL group compared to the CON group. The relative abundance of 24 genera significantly increased and 79 genera significantly decreased in the ABX group compared to the CON group. The relative abundance of 26 genera significantly increased and 35 genera significantly decreased in the ABX group compared to the SAL group. Compared to the CON group, 9 identical genera had significantly higher relative abundance in both the SAL and ABX groups, accounting for 34.62% of the SAL group and 37.50% of the ABX group, respectively. Compared to the CON group, 25 identical genera had significantly lower relative abundance in both the SAL and ABX groups, accounting for 62.50% of the SAL group and 31.65% of the ABX group, respectively.

The linear discriminant analysis (LDA) showed that three phyla (e.g., *Actinobacteriota*, *Bacteroidota*, and *Desulfobacterota*) and nine genera (e.g., *Clostridia_UCG_014_unclassified*, *Faecalibacterium*, *Lactobacillus*, *Eubacterium_coprostanoligenes_group_unclassified*, *Prevotellaceae_UCG_001*, *Megasphaera*, *HT002*, *Barnesiella*, and *Clostridium*) were significantly different between groups (*P* < 0.05) (Supplementary Figure S3). The phylum *Bacteroidota* and the genus *Prevotellaceae_UCG_001* had the highest LDA scores at the phylum and genus level, respectively, suggesting that they had a greater influence on the differences between groups.

### 3.3 *Salmonella Typhimurium* infection affects polyunsaturated fatty acid metabolism in intestinal tissues

A total of 130 oxidized lipid metabolites were detected from 18 cecum tissue samples, and 71 metabolites were retained after preprocessing (Supplementary Table S9). These included thirty-seven mid-ARA metabolites, eight docosahexaenoic acid (DHA) metabolites, eight linoleic acid (LA) metabolites, five dihomo-γ-linolenic acid (DGLA) metabolites, five eicosapentaenoic acid (EPA) metabolites, two α-linolenic acid (ALA) metabolites, one docosatetraenoic acid (DTA) metabolite, and one gamma-linolenic acid (GLA) metabolite.

**FIGURE 4:**
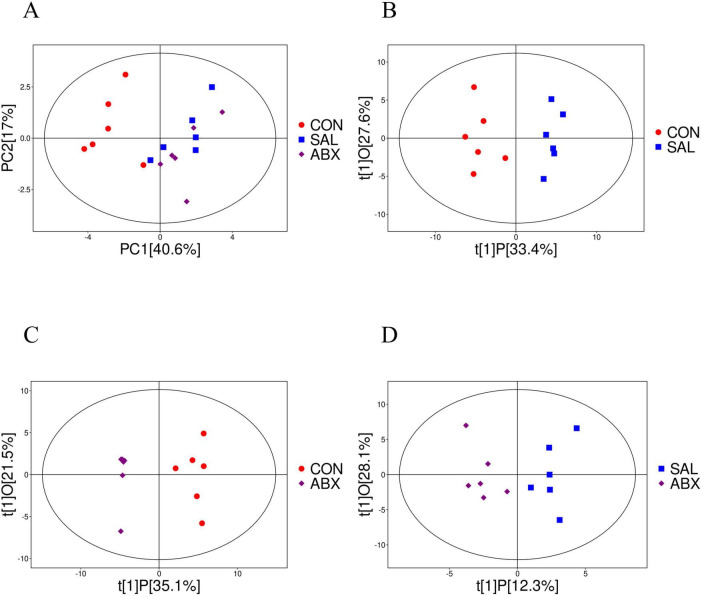
Analysis of PCoA and OPLS-DA between the CON, SAL, and ABX groups. **(A)** Scatterplot of principal component analysis (PCA) scores for the full sample in each group. **(B–D)** Scatterplot of OPLS-DA model scores between groups. The horizontal coordinate represents the predicted principal component scores of the first principal component, demonstrating the differences between sample groups, whereas the vertical coordinate represents the orthogonal principal component scores, demonstrating the differences within the sample groups, where each scatter represents one sample and the scatter shapes and colors indicate the different experimental groups.

**FIGURE 5:**
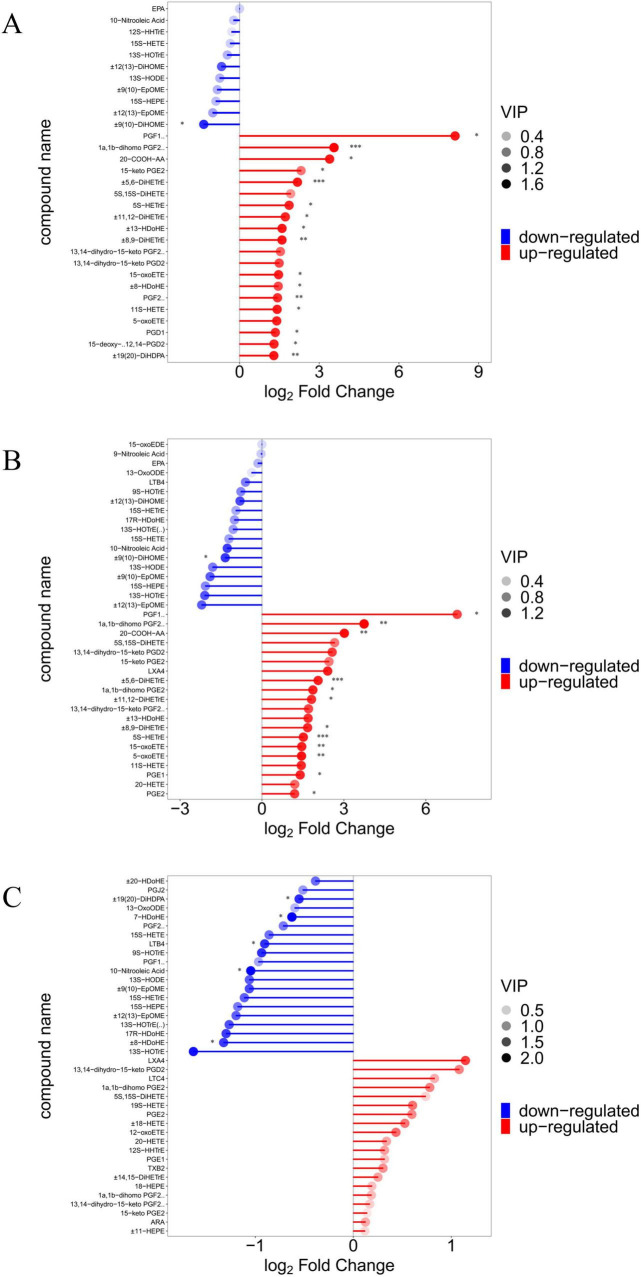
Demonstration of differences in oxygenated lipid metabolites between groups. **(A–C)** Matchstick analysis for three comparison groups (SAL vs. CON, ABX vs. CON, and ABX vs. SAL). Horizontal coordinates show logarithmically converted multiples of change, with point color shades representing the size of the variable importance in the projection (VIP) value. “*” indicates a significant difference in statistics (*P < 0.05, **P < 0.01, and ***P < 0.001).

The results of principal component analysis (PCA) for the 71 metabolites showed that the samples from the ABX and SAL groups were distinct from the samples from the CON group, and the samples from the ABX and SAL groups were not completely separated (Figure 4; Supplementary Figure S4). The multiplicity of differences between the top 20 metabolites that were increased and the top 20 metabolites that were decreased in each comparison group (SAL vs. CON, ABX vs. CON, and ABX vs. SAL) are shown in Figures 5A–C. The Z-score for each comparison group is shown in Supplementary Figure S5. The results of the cluster analysis of metabolites in each group are shown in Supplementary Figure S6. The above results visually indicated that the content of multiple oxidized lipid metabolites was altered in the cecum tissues of the SAL group.

### 3.4 Overall enhancement of arachidonic acid metabolism in chicken cecum tissue caused by *Salmonella Typhimurium* infection

In this experiment, the groups were screened for differential metabolites with a *p*-value of less than 0.05 (Supplementary Table S10). The results showed that 26 metabolites were significantly higher (*P* < 0.05) and one significantly lower ( ± 9(10)-DiHOME) in the SAL group compared to the CON group (*P* < 0.05), for a total of 27 metabolites that were significantly changed. These included fifteen ARA metabolites, three DGLA metabolites, six DHA metabolites, one LA metabolite, and one DTA metabolite. Of the fifteen ARA differential metabolites in the SAL group, two were COX metabolic pathway products (e.g., PGF2α and 15-keto-PGE_2_), two belonged to the LOX pathway (e.g., LXA4 and 15-OxoETE), and six belonged to the CYP metabolic pathway (e.g., ± 11(12)-EET, ± 8(9)-EET, ± 14,15-DiHETrE, ± 11,12-DiHETrE, ± 8,9-DiHETrE, and ± 5,6-DiHETrE). The results are displayed in the Kyoto Encyclopedia of Genes and Genomes (KEGG) pathway enrichment analysis plot (Supplementary Figure S7A).

**FIGURE 6:**
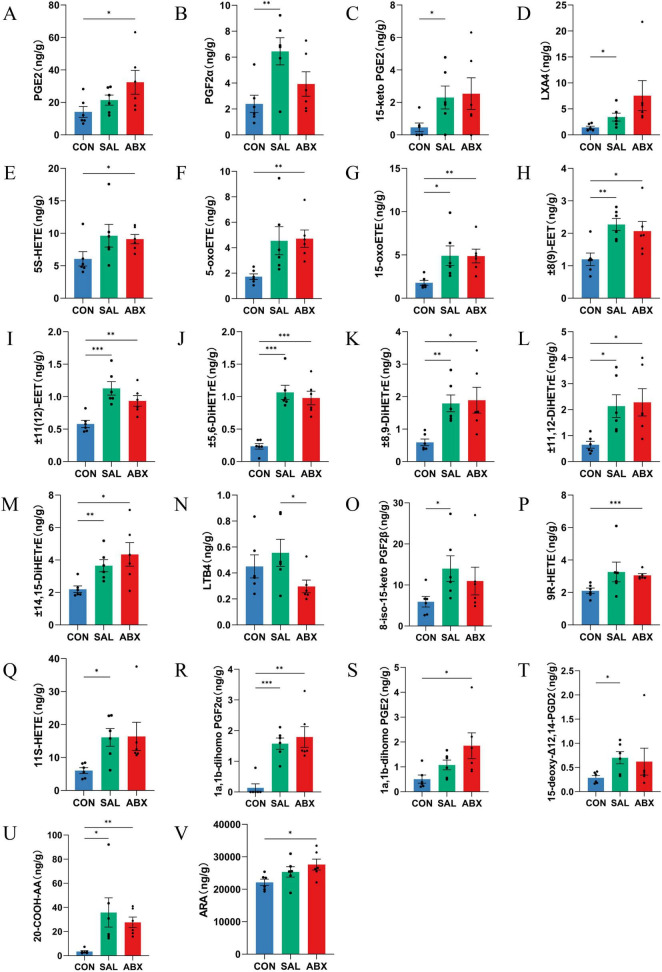
Analysis of between-group differences in ARA metabolites. **(A–V)** Metabolites of ARA. “*” indicates a significant difference in statistics (*P < 0.05, **P < 0.01, and ***P < 0.001).

There were 22 metabolites with significantly higher levels (*P* < 0.05) and one with a significantly lower level ( ± 9(10)-DiHOME) in the ABX group compared to the CON group (*P* < 0.05), for a total of 23 metabolites that were significantly changed. These included 15 ARA metabolites, three DGLA metabolites, one DHA, one LA metabolite, and one DTA metabolite. Of the 15 ARA differential metabolites in the ABX group, one was a product of the COX metabolic pathway product (e.g., PGE_2_), three belonged to the LOX pathway (e.g., 5S-HETE, 5-oxoETE, and 15-OxoETE), and six belonged to the CYP metabolic pathway (e.g., ± 11(12)-EET, ± 8(9)-EET, ± 14,15-DiHETrE, ± 11,12-DiHETrE, ± 8,9-DiHETrE, and ± 5,6-DiHETrE). The results are displayed in the KEGG pathway enrichment analysis (Supplementary Figure S7B). Fifteen of the differential metabolites were found to be identical in the SAL and ABX groups, accounting for 55.56 and 68.2% of these groups, respectively. Differences in ARA metabolites between groups are presented as bar graphs (Figure 6).

### 3.5 Spearman analysis results between oxidized lipid metabolites, intestinal flora, and inflammatory factors

**FIGURE 7:**
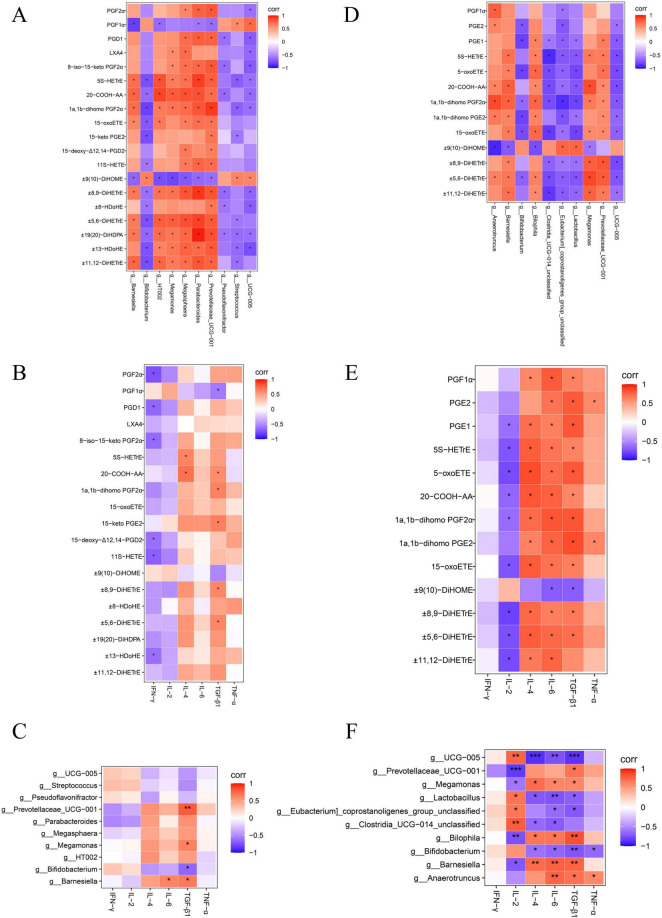
Heatmap of correlation coefficient matrix for the SAL and ABX groups. **(A–C)** Analysis of Spearman’s correlation between oxidized lipid metabolites, gut microbes, and inflammatory factors in the SAL group. **(D–F)** Analysis of Spearman’s correlation between oxidized lipid metabolites, gut microbes, and inflammatory factors in the ABX group. Red indicates a positive correlation, and purple indicates a negative correlation. “*” indicates a significant difference in statistics (*P < 0.05, **P < 0.01, and ***P < 0.001).

The relative mRNA levels of IL-4 and transforming growth factor-beta 1 (TGF-β1) were increased in the SAL group compared with the CON group (*P* < 0.05), and there was a trend toward higher levels of IL-6 (*P* > 0.05) (Supplementary Figure S8). Relative mRNA levels of interferon-gamma (IFN-γ), IL-4, TGF-β1, and IL-6 were increased (*P* < 0.05), and IL-2 levels were decreased (*P* < 0.05) in the ABX group compared with the CON group (Supplementary Figure S8). Tumor necrosis factor-alpha (TNF-α), IL-4, TGF-β1, IFN-γ, and IL-6 levels were higher (*P* < 0.05) in the ABX group than in the SAL group, and IL-2 and interleukin 1β (IL-1β) levels were lower (*P* < 0.05) in the ABX group than in the SAL group (Supplementary Figure S8). The results of the “Spearman” analysis between oxidized lipid metabolites, intestinal flora, and inflammatory factors are shown in Figure 7 and Supplementary Tables S11, S12.

### 3.6 *Salmonella Typhimurium* infection activates the cyclooxygenase metabolic pathway of arachidonic acid

**FIGURE 8:**
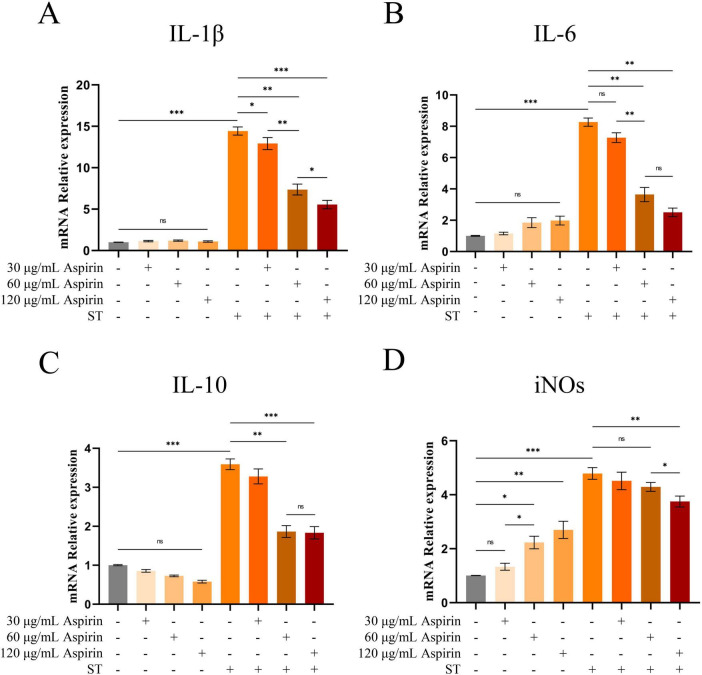
Changes in IL-1, IL-6, IL-10 and iNOs in HD11 cells. **(A–D)** The relative mRNA expression levels of IL-1β, IL-6, IL-10 and iNOs in HD11 cells are indicated, respectively. “*” indicates a significant difference in statistics (*P < 0.05, **P < 0.01, and ***P < 0.001).

The results of cellular experiments showed that aspirin pretreatment did not significantly affect the relative expression levels of IL-1β, IL-6, and IL-10 mRNA in HD11 cells compared with the untreated group (Figures 8A–C). However, the iNOs were significantly increased in HD11 cells after treatment with 60 and 120 μg/mL aspirin and strengthened with the increase of aspirin concentration (Figure 8D). Compared to the untreated group, relative mRNA levels of IL-1β, IL-6, IL-10, and iNOs were increased in HD11 cells after *S. Typhimurium* infection (*P* < 0.001). Whereas aspirin pretreatment reduced this effect in a dose-dependent manner. The results of cellular experimental modeling and drug concentration screening are shown in Supplementary Figures S9, S10.

### 3.7 *Salmonella Typhimurium* infection activates the cyclooxygenase metabolic pathway of arachidonic acid

**FIGURE 9:**
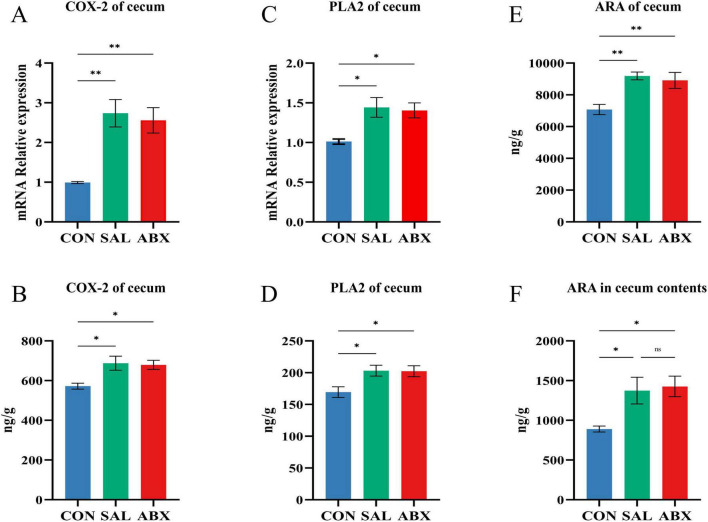
Changes of arachidonic acid and its key enzymes of metabolic pathway in the cecum. (**A**–**D**) COX-2 and PLA2 content and relative mRNA expression levels in cecum tissues. **(E,F)** ARA content in cecum tissues and cecum contents. “*” indicates a significant difference in statistics (*P < 0.05, **P < 0.01, and ***P < 0.001).

The levels of COdX-2 and PLA2 in cecum tissues were increased in both the SAL and ABX groups after *S. Typhimurium* infection compared to the CON group (*P* < 0.05 or *P* < 0.01) and did not differ between either the SAL and ABX groups (Figures 9A–D). Compared to the CON group, the levels of ARA in cecum tissues and cecum contents were increased in both the SAL and ABX groups after *S. Typhimurium* infection (*P* < 0.05), and there was no difference between the SAL and ABX groups (Figures 9E,F).

**FIGURE 10:**
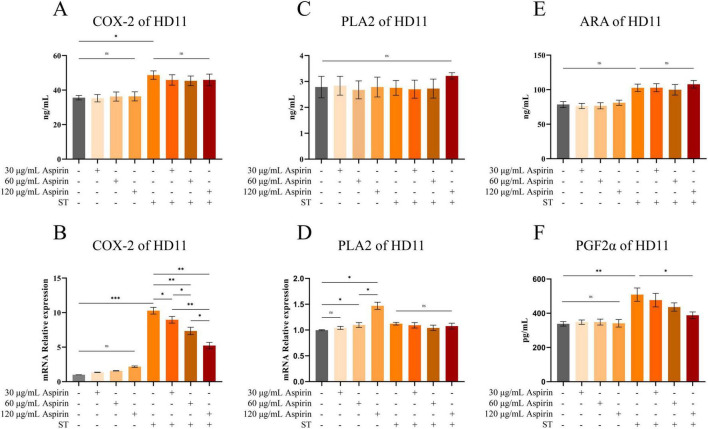
Changes of arachidonic acid and its key enzymes of metabolic pathway in HD11 cells. **(A,C,E,F)** The contents of COX-2, PLA2, ARA, and PGF2α in HD11 cells are indicated, respectively. **(B,D)** The relative mRNA expression levels of COX-2 and PLA2 in HD11 cells are indicated, respectively. “*” indicates a significant difference in statistics (*P < 0.05, **P < 0.01, and ***P < 0.001).

Compared to the untreated group, aspirin pretreatment had no significant effect on the levels of COX-2 in HD11 cells (Figures 10A,B), but the relative mRNA expression level of PLA2 was significantly increased when the concentration reached 60 and 120 μg/mL (*P* < 0.05) with a dose-dependent trend (Figure 10D). Compared to the untreated group, aspirin pretreatment had no significant effect on the levels of PLA2, PGF2α, and ARA in HD11 cells (Figures 10C,E,F).

Compared to the untreated group, the relative mRNA expression level of COX-2 in HD11 cells was increased after *S. Typhimurium* infection (*P* < 0.001), whereas aspirin pretreatment significantly reduced this effect (Figure 10B) (*P* < 0.05 or *P* < 0.01). Meanwhile, Elisa’s results showed that COX-2 content in HD11 cells was increased after *S. Typhimurium* infection (*P* < 0.05). However, aspirin pretreatment could not inhibit the increased COX-2 content induced by infection (Figure 10A). Compared to the untreated group, PGF2α content in HD11 cells was increased after *S. Typhimurium* infection (*P* < 0.05). However, aspirin pretreatment (120 μg/mL) reduced the infection-induced increases in PGF2α content (Figure 10F) (*P* < 0.05). Compared to the untreated group, the levels of ARA and PLA2 in HD11 cells did not change significantly after *S. Typhimurium* (Figures 10C,E).

## 4 Discussion

Intestinal inflammation caused by avian salmonellosis is necessary for *Salmonella* colonization and survival (Winter et al., 2010). Arachidonic acid (ARA) metabolites are an important and widely acting class of inflammatory mediators (Huang et al., 2021). Therefore, the relationship between arachidonic acid metabolites and enteritis caused by *Salmonella* infection in poultry, which is for the prevention and treatment of the disease.

PLA2 and COX-2 are key enzymes for ARA production and metabolism (Murakami et al., 2017; Pang et al., 2022), respectively. *S. Typhimurium* infection caused increased levels of PLA2 and COX-2 in the intestinal tissues of Wenchang chickens, which may be the direct cause of the increased levels of arachidonic acid and its metabolites in the cecum tissues. A number of studies have shown that ARA metabolites (e.g., prostaglandins, leukotrienes, and lipoxins) mediate inflammatory responses in the intestine via different types of G-protein-coupled receptors (Stenson, 2014; Kawahara et al., 2015; Yokomizo et al., 2018; Nagatake and Kunisawa, 2019). For example, PGE_2_ signals through prostaglandin E receptor 2 (EP2) on neutrophils and tumor-associated fibroblasts, which promotes inflammation through multiple steps to form the tumor microenvironment in colorectal cancer (Aoki and Narumiya, 2017) and induces mast cell activation through the EP3 receptor signaling pathway, which enhances vascular permeability and promotes acute inflammation (Morimoto et al., 2014). PGF2α levels are significantly increased in patients with ulcerative enteritis, which is alleviated by the use of PLA2 inhibitors (Lauritsen et al., 1987). 5-OxoETE (precursor of 5(S)-HETE) promotes the basophil migratory response (Iikura et al., 2005), and is involved in neuroinflammatory activities (Wang et al., 2023). LXA4 facilitates the reduction in chronic inflammation, inhibits the production of PGE_2_ and LTs, and suppresses the production of pro-inflammatory factors IL-6 and TNF-α (Das, 2021). 15-OxoETE can activate anti-inflammatory Nrf2 signaling and inhibit the NF-κB-mediated pro-inflammatory pathway (Snyder et al., 2015). EETs (e.g., 8,9-EET, 11,12-EET) prevent leukocyte adherence to the vascular wall through inhibition of the transcription factors NF-κB and IκB kinase and play an important non-vasodilatory role in vascular inflammation (Node et al., 1999). EETs can also inhibit the activation of NOD-like receptor thermal protein domain associated protein three inflammatory vesicles by suppressing calcium overload and reactive oxygen species production in macrophages, thereby facilitating the treatment of acute lung injury (Luo et al., 2020). It is thus evident that arachidonic acid metabolites have both anti-inflammatory and pro-inflammatory effects. We speculate that arachidonic acid metabolites are key substances mediating enteritis caused by *Salmonella* infection.

Prostaglandins such as PGE2 and PGF2α are metabolized by ARA by the COX-2 pathway (Li et al., 2024). We found that *S. Typhimurium* infection significantly increased the levels of COX-2 and PGF2α in HD11 cells and promoted the mRNA expression of inflammatory factors (IL-1β, IL-6, and iNOs). Furthermore, a cyclooxygenase inhibitor (aspirin) significantly inhibited the inflammatory response induced by *S. Typhimurium* infection. This validates our conjecture, suggesting that the ARA cyclooxygenase metabolic pathway mediates the inflammatory response upon *S. Typhimurium* infection. Unfortunately, this study did not examine how ARA metabolites mediate the development and progression of enteritis infected by *Salmonella* in chickens. This is because many of the metabolites have not been studied in vivo or in vitro in poultry, and some of the metabolites (e.g., EETs and PGE_2_) are rapidly degraded to low effectors in the organism, making it difficult to control the experimental variables (Zhou et al., 2017). Therefore, it is necessary to explore the research conditions further, find suitable experimental methods for validation, and gradually explore the mechanism of action of the ARA metabolites in *Salmonella-*induced intestinal inflammation in the future.

Gut microbiota composition is one of the most important factors affecting animal intestinal immunity and serves as a barrier against invasion by pathogenic microorganisms. To explore the resistance of chickens’ intestinal flora to Salmonella infection, we used antibiotic treatments in the ABX group to simulate a state of intestinal flora dysbiosis. Infection with *S. Typhimurium* significantly reduced the number of microorganisms, the flora abundance, and the species diversity in the cecum of Wenchang chickens. This is similar to the changes in the intestinal flora of laying hens after *Salmonella* infection (Mon et al., 2020). In addition, the relative contents of cytokines such as IL-4, IL-6, TGF-β1, and IFN-γ were significantly higher in the ABX group than in the SAL group. The above results suggest that Salmonella infection leads to intestinal flora dysbiosis, while the lack of intestinal flora further exacerbates the inflammatory response caused by Salmonella infection. Notably, the abundance of many beneficial bacteria (*Bifidobacterium*, *Lactobacillus*, and *Odoribacter*) was significantly reduced in the SAL and ABX groups. Many studies have confirmed the beneficial effects of Bifidobacterium in animals. It prevents diarrhea (Videlock and Cremonini, 2012), alleviates the symptoms of irritable bowel syndrome (Lewis et al., 2020), prevents colorectal cancer (Bahmani et al., 2019), alleviates clinical signs in patients with ulcerative colitis (Ishikawa et al., 2011), and regulates and maintains the structure of the intestinal flora (Luo et al., 2018). Lactobacillus is a group of beneficial intestinal bacteria that can prevent Clostridium difficile-infection-associated diarrhea (Goldenberg et al., 2017), facilitate intestinal inflammation (Ahl et al., 2016), and regulate immune-related molecules to enhance intestinal barrier function (Schlee et al., 2008; Terada et al., 2020).

The Spearman analysis showed a significant positive correlation between the relative abundance of *Bifidobacterium* and *Lactobacillus* and the relative mRNA expression of IL-2 in intestinal tissues (*P* < 0.05). IL-2 is a cytokine essential for the growth and survival of regulatory T cells (Tregs) in peripheral lymphoid tissues. Tregs are essential for the maintenance of peripheral tolerance and for the control of persistent inflammation and autoimmunity (Graßhoff et al., 2021). However, the relative mRNA content of IL-2 in the intestinal tissues of Wenchang chickens tended to decrease after S. Typhimurium infection. This suggests that avian gut microbiota, especially beneficial bacteria, potentially regulate intestinal immunity. However, *Salmonella* infection reduces the abundance of beneficial flora, which in turn reduces the immune function of the intestine, leading to the overgrowth of certain inflammation-associated genera in the avian intestinal tract, further aggravating intestinal inflammation. In summary, probiotics or other beneficial substances can be appropriately added to feed to regulate the flora structure and improve resistance to harmful microorganisms (Al-Khalaifa et al., 2019; Khan et al., 2020; Neveling and Dicks, 2021). For example, *Odoribacter* has shown promise as a next-generation probiotic that improves glucose tolerance and obesity-associated inflammation (Huber-Ruano et al., 2022). *Lactofructose* attenuated colitis symptoms by upregulating *Muribaculum* abundance (Hiraishi et al., 2022). An inevitable problem in this study was that it was not possible to experimentally verify these results. This is because the gut microbial system is too complex to control these variables.

In summary, this study found that infection with *S. Typhimurium* resulted in disturbed intestinal flora and a significant activation of ARA metabolism in Wenchang chickens. It also reported some ARA metabolites to be involved in the induction of inflammation. Given that the inflammatory intestinal environment is conducive to colonization and invasion by *Salmonella*, it is suggested that modulating ARA metabolism in intestinal tissues may be a potential approach to control enteritis caused by *Salmonella* infection.

## 5 Conclusion

This study found that *S. Typhimurium* infection induced levels of several arachidonic acid metabolites in chicken cecum tissues. In HD11 cells, a cyclooxygenase inhibitor significantly reduced the inflammatory response induced by *S. Typhimurium* infection. In addition, we observed the presence of disturbed cecal intestinal flora in the infected group, but the effect of this phenomenon on ARA metabolism was not significant. The above results suggest that *S. Typhimurium* can mediate the onset and development of intestinal inflammation by activating the chicken ARA cyclooxygenase metabolic pathway. These findings may provide new insights into how *Salmonella* induces enteritis in chickens. Based on this study, potential new mechanisms for combating enteritis caused by *Salmonella* infection in poultry can be further explored.

## Data Availability

The datasets presented in this study can be found in online repositories. The names of the repository/repositories and accession number(s) can be found at: https://www.ncbi.nlm.nih.gov/, PRJNA1027078.

## Footnotes

1
https://cran.r-hub.io/bin/windows/base/

2
https://www.medcalc.org/

3
https://cran.r-hub.io/bin/windows/base/old/3.4.4/

4
https://search.r-project.org/R/refmans/stats/html/00Index.html

5
https://cran.r-project.org/web/packages/vegan/index.html
