# Supplementary material for: Effects of Salmonella Typhimurium infection on intestinal flora and intestinal tissue arachidonic acid metabolism in Wenchang chickens
Source: Front Microbiol. 2025 Jan 24;16:1514115. doi: 10.3389/fmicb.2025.1514115 (PMC11803450; doi:10.3389/fmicb.2025.1514115)
Supplement: Supplementary file 1 [file Data_Sheet_1.zip › Supplementary File(s)/Supplementary_Figures.docx]

Supplementary Material

Effects of Salmonella Typhimurium infection on intestinal flora and intestinal tissue arachidonic acid metabolism in Wenchang chickens

Shenghong Chen, Yaochen Xie, Dingqian Guo, Tiansen Li, Zhen Tan, Xuhua Ran* and Xiaobo Wen*

*** Correspondence:** Xiaobo Wen: xiaobo_wen@hainanu.edu.cn

# Supplementary Figures


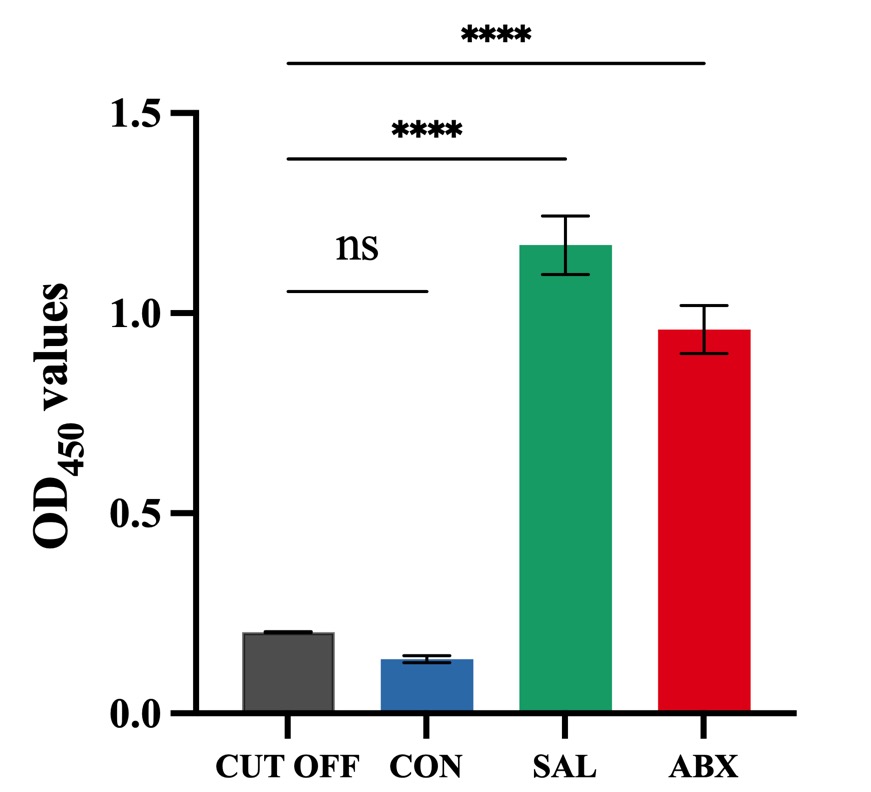


Supplementary Figure S1. Detection results of *S. Typhimurium* infection models. The CUT OFF value is 0.2 and higher than the CUT OFF value indicates that Salmonella was detected in the sample and vice versa. “*” indicates a significant difference in statistics (**P* < 0.05, ***P* < 0.01, and *** *P* < 0.001).


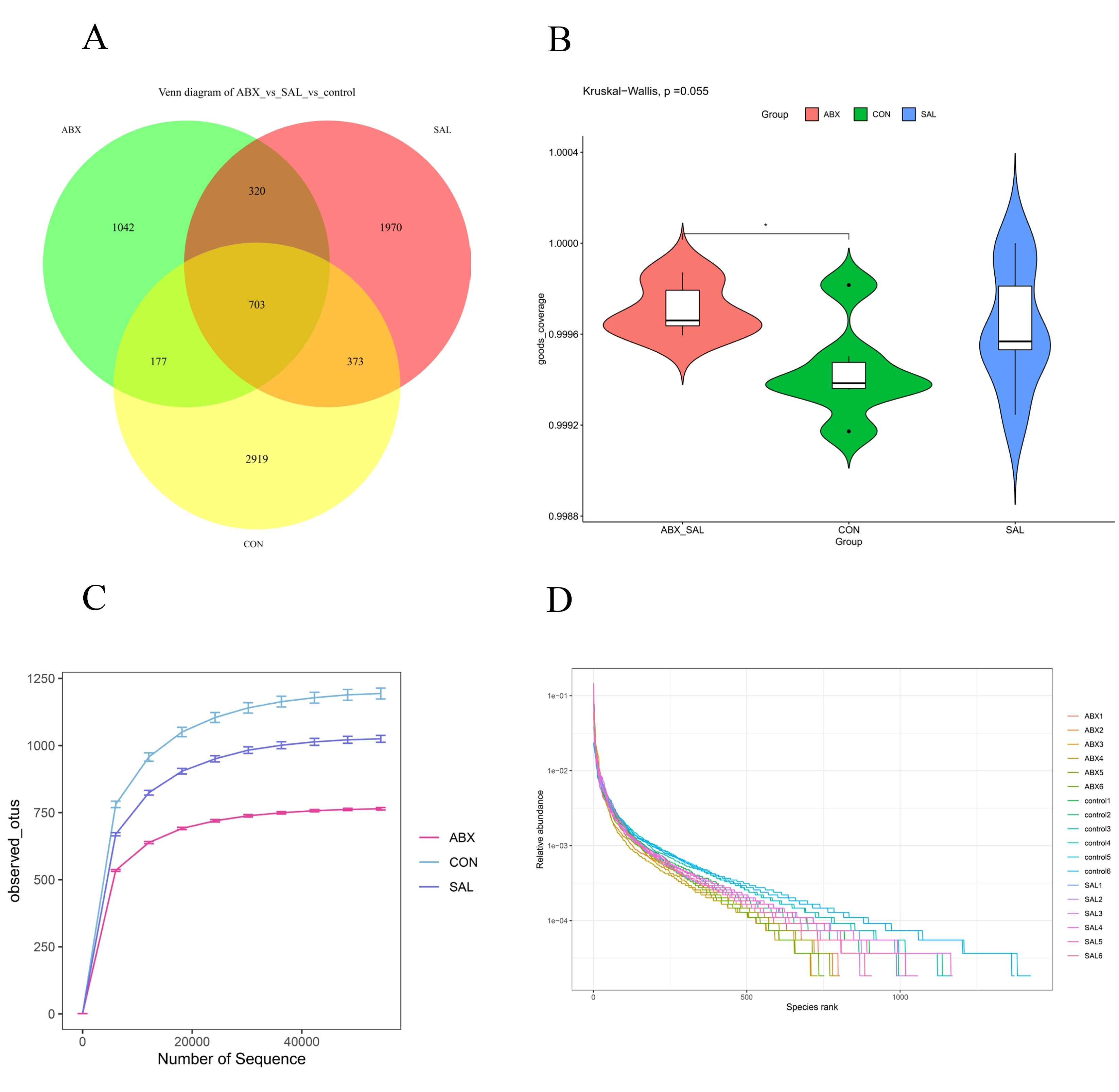


Supplementary Figure S2. DNA sequence data analysis. (**A**) Venn diagram. The numbers in the figure show the unique or shared OTUs in each group. (**B**) Graph showing the difference in Goods_coverage between groups. Dilution curve (**C**) and abundance rank curve (**D**) of ASV. “*” indicates a significant difference in statistics (**P* < 0.05, ***P* < 0.01, and *** *P* < 0.001).


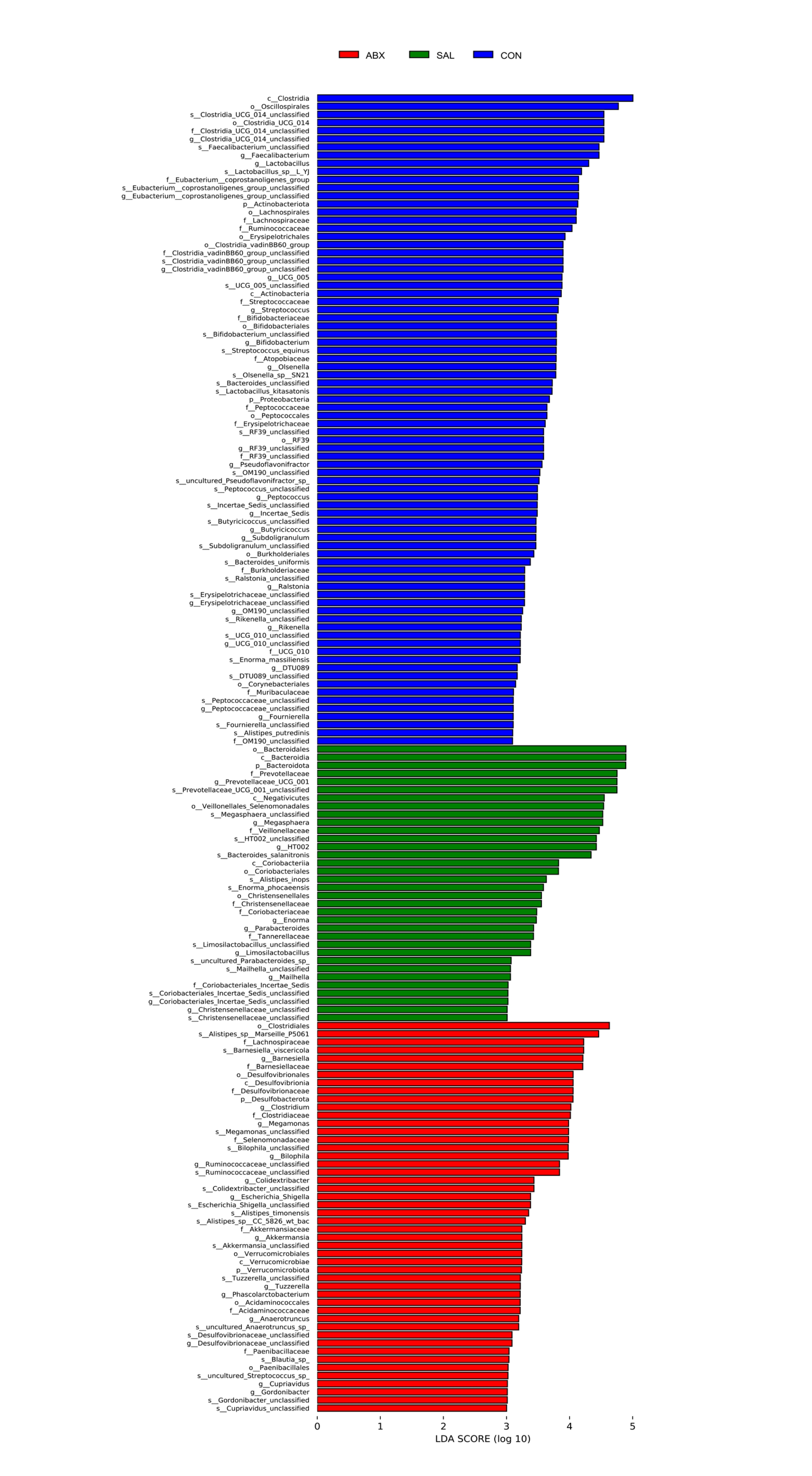


Supplementary Figure S3. Significant differences in bacterial taxa between groups were determined by linear discriminant analysis and effect size (LEfSe). LDA scores were calculated for bacterial taxa that were differentially enriched among different groups.


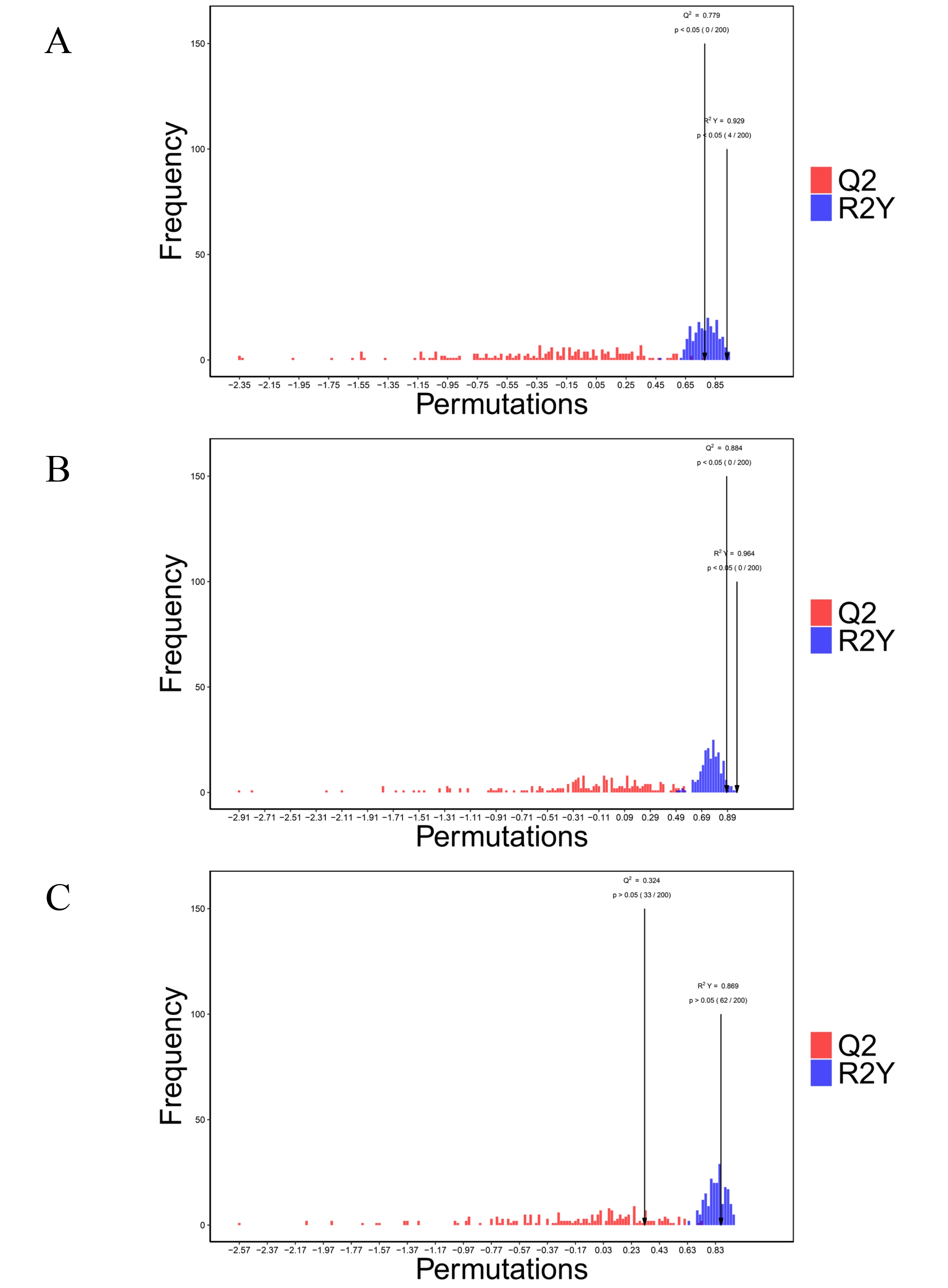


Supplementary Figure S4. Histogram of replacement test results for OPLS-DA model. The horizontal coordinate represents the accuracy of the replacement test random model, and the vertical coordinate represents the number of random models. The red bar represents the number of occurrences of Q2 value obtained from the replacement test, and the blue bar represents the number of occurrences of R2Y value obtained from the replacement test. The p-value is the ratio of the number of stochastic models in the permutation test that outperforms the original model divided by the number of all stochastic models in the permutation test. The model is considered optimal when the p-value is less than 0.05.


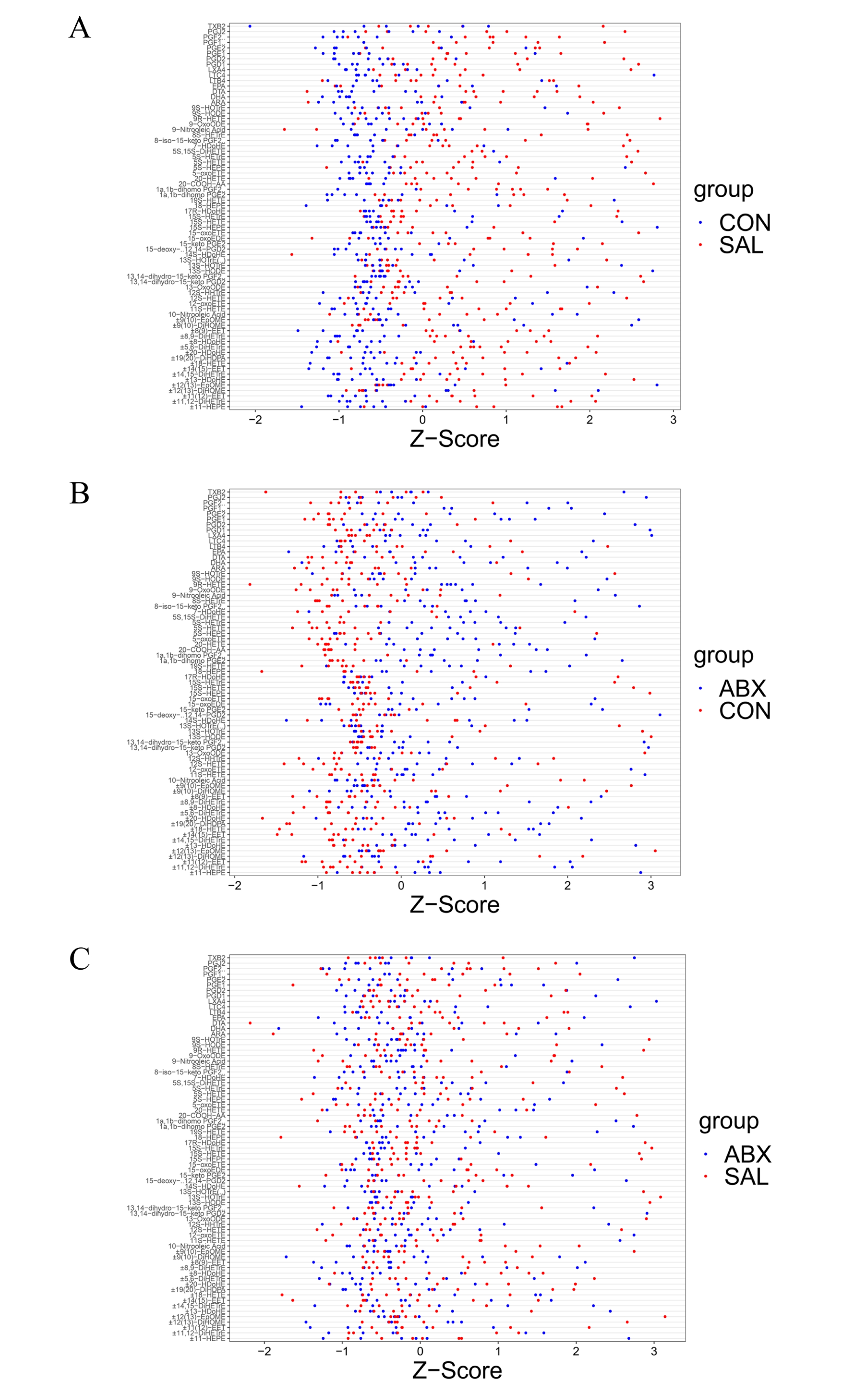


Supplementary Figure S5. Relative values of the Z-score for the three comparison groups (SAL vs. CON, ABX vs. CON, and ABX vs. SAL). Only the top 20 substances with up- and down-regulated multiples in each comparison group are shown here. The abscissa represents the Z-score, the ordinate represents the metabolites, and the points of different colors represent the samples of various groups.


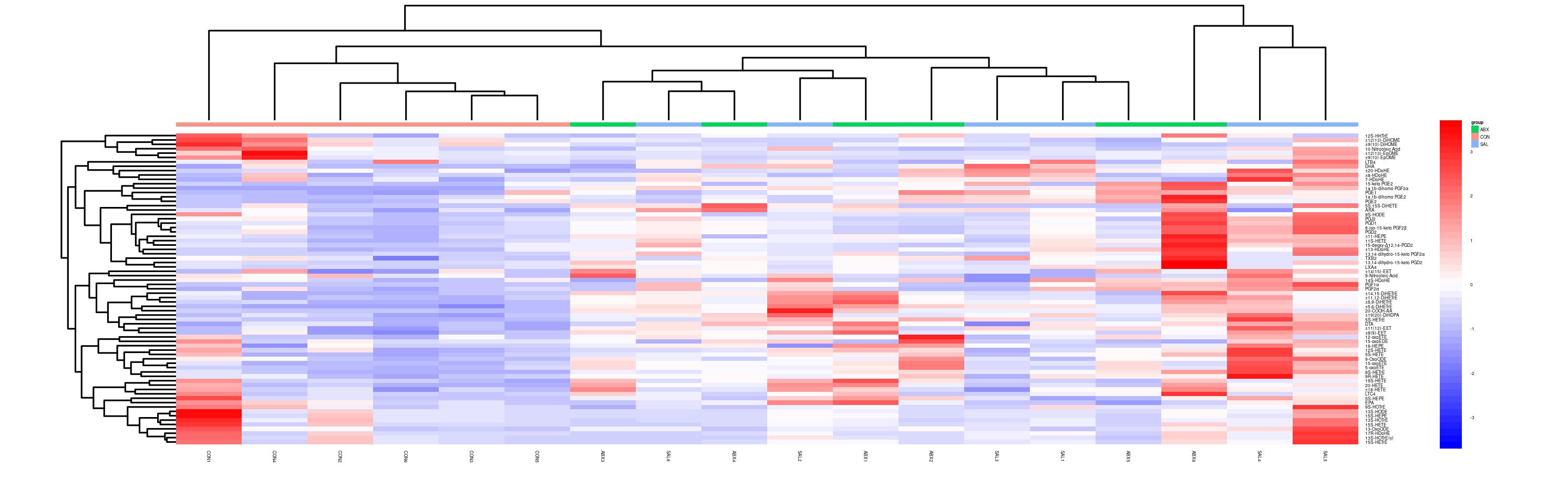


Supplementary Figure S6. Results of metabolite cluster analysis. The horizontal coordinates in the graph represent different experimental groups, the vertical coordinates represent the metabolites compared in the group, and the colored blocks in various positions represent the relative expression of the metabolites in the corresponding positions. Red indicates that the substance is highly expressed in the group in which it is contained, and blue means that the substance is lowly expressed in the group in which it is included.


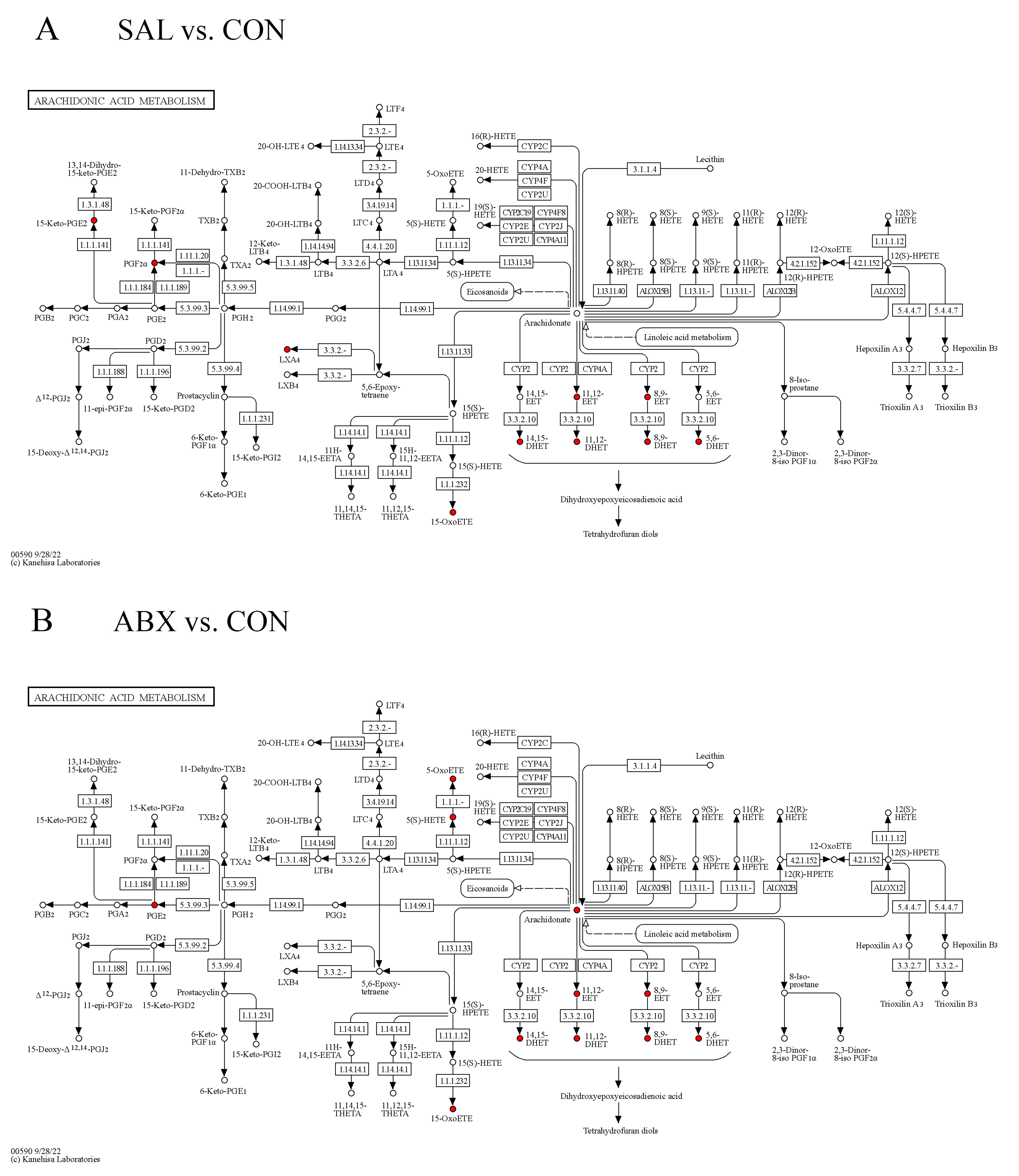


Supplementary Figure S7. KEGG enrichment pathway map of the differential metabolites in the SAL and ABX groups. (**A**) KEGG enrichment analysis of the differential metabolites in the SAL group. (**B**) Results of KEGG enrichment analysis of the differential metabolites in the ABX group. Red represents significant metabolite upregulation.


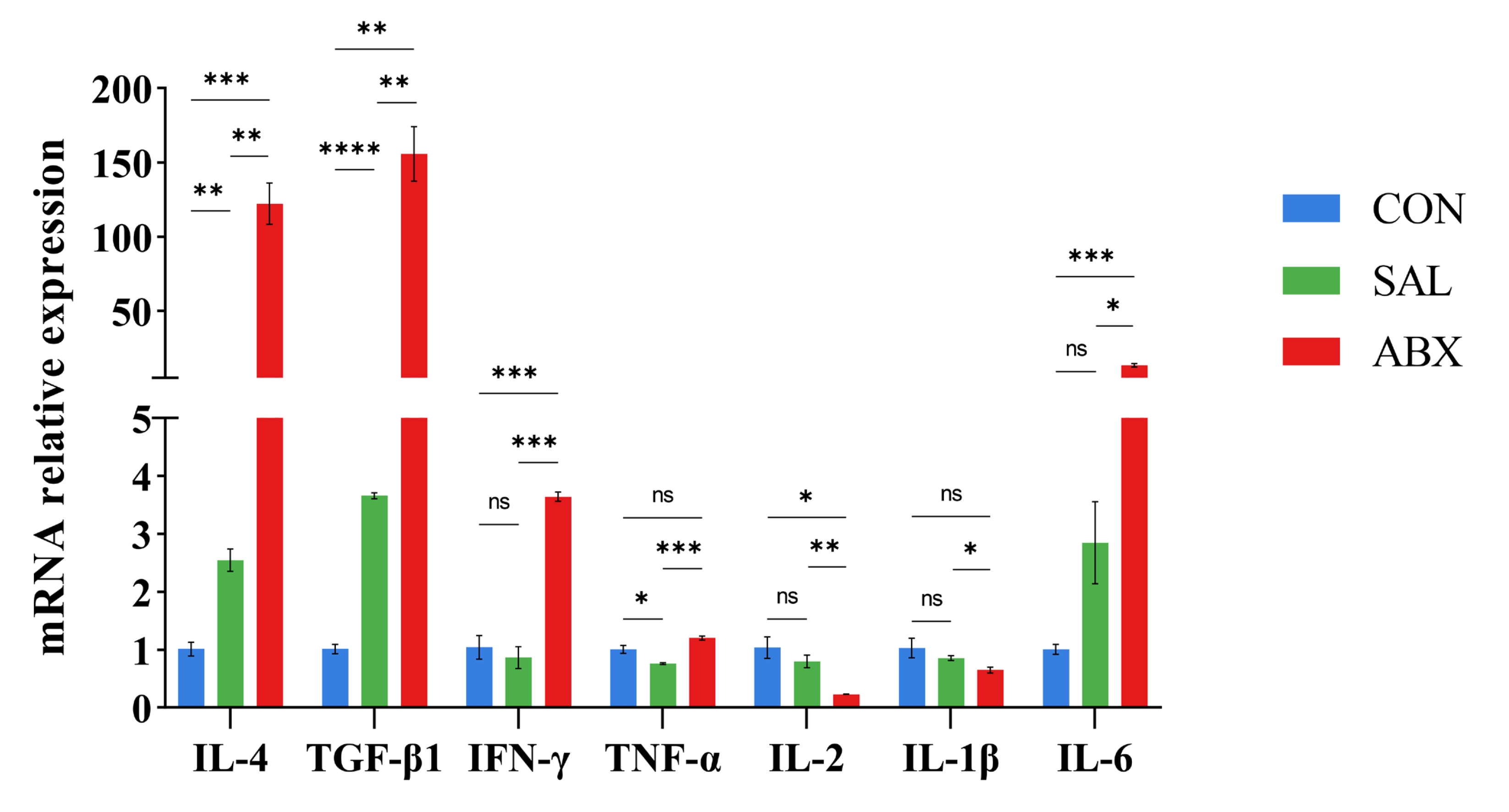


Supplementary Figure S8. Relative mRNA levels of inflammatory factors in cecum tissue. “*” indicates a significant difference in statistics (**P* < 0.05, ***P* < 0.01, and *** *P* < 0.001).


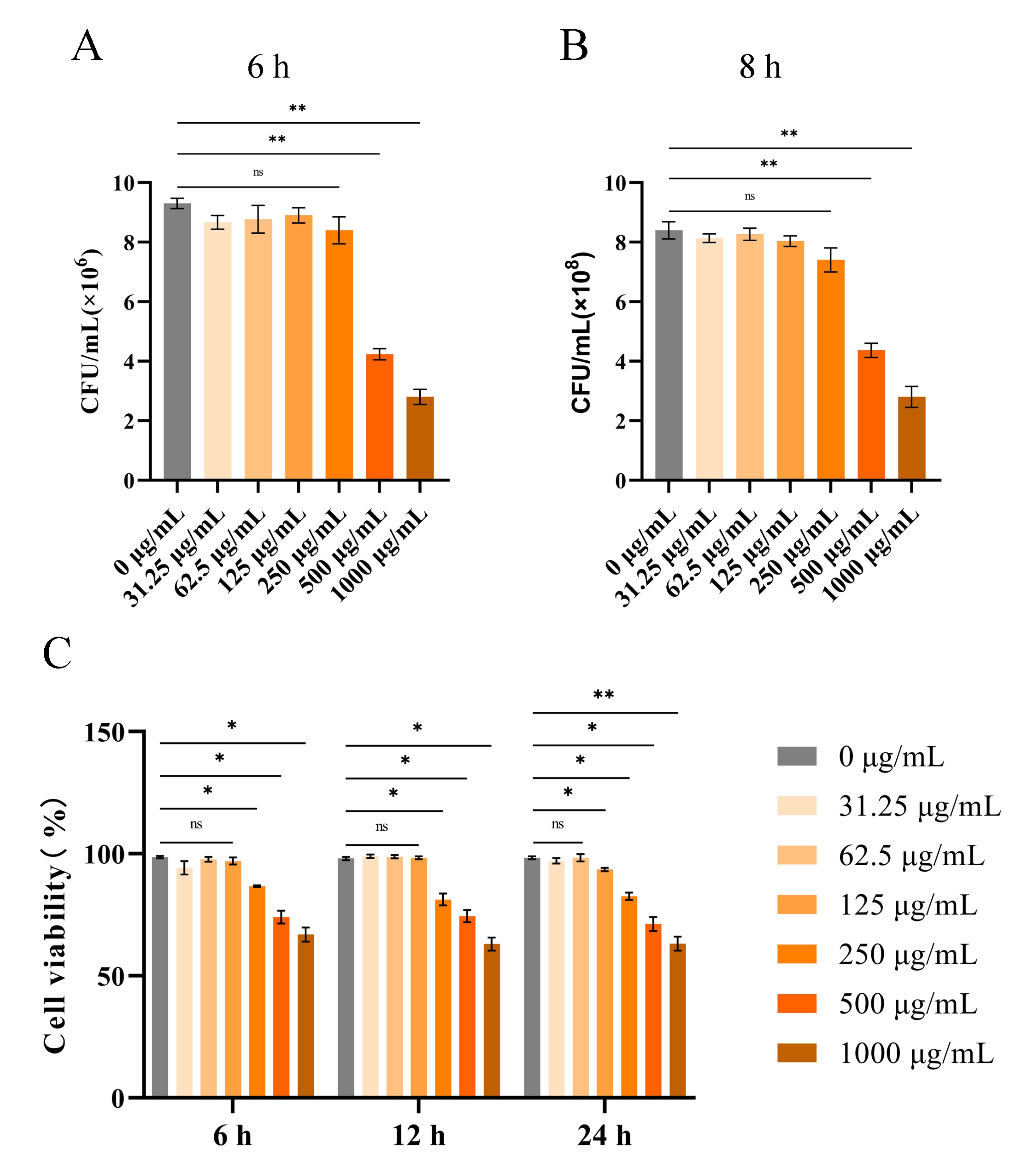


Supplementary Figure S9. Effect of different concentrations of aspirin at different treatment times on the activity of HD11 cells and *S. Typhimurium*. “*” indicates a significant difference in statistics (**P* < 0.05, ***P* < 0.01, and *** *P* < 0.001).


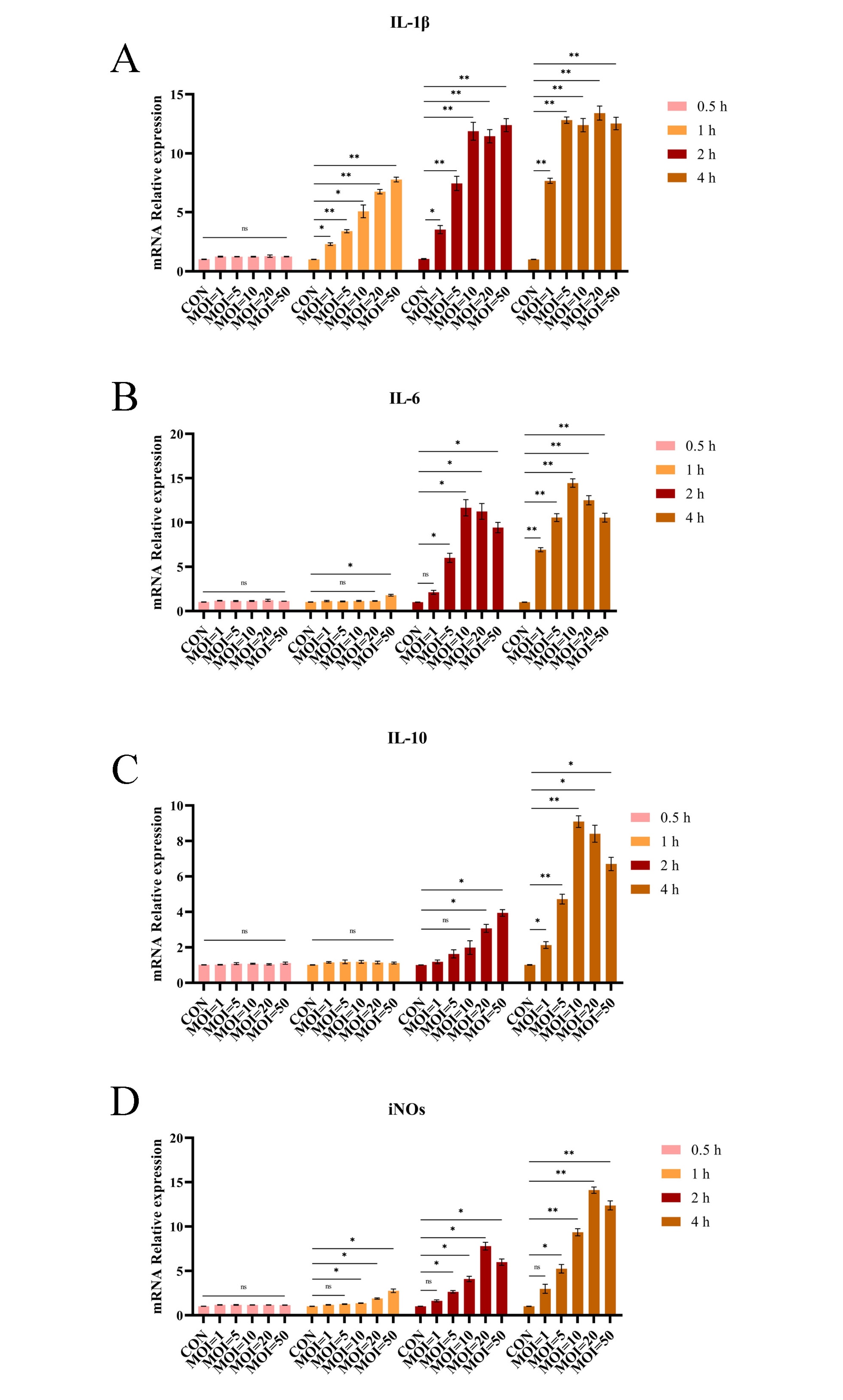


Supplementary Figure S10. Effect of different infection multiplicities of *S. Typhimurium* on inflammation-related gene expression in HD11 cells within a time gradient. “*” indicates a significant difference in statistics (**P* < 0.05, ***P* < 0.01, and *** *P* < 0.001).
