# Supplementary material for: Effects of Salmonella Typhimurium infection on intestinal flora and intestinal tissue arachidonic acid metabolism in Wenchang chickens
Source: Front Microbiol. 2025 Jan 24;16:1514115. doi: 10.3389/fmicb.2025.1514115 (PMC11803450; doi:10.3389/fmicb.2025.1514115)
Supplement: Supplementary file 1 [file Data_Sheet_1.zip › Supplementary File(s)/Supplementary Tables/Table S1-qRT-PCR primer information.docx]

Table 1. Primer sequences of chicken cytokines used for the quantitative real-time PCR.

| RNA target | Primer sequence (5' to 3') | Accession no. | Length of product(bp) |
| --- | --- | --- | --- |
| COX-2 | F: 5' CTGTTGGGCAGGAGGTGTTTGG 3' | NM_001167718 | 126 |
|  | R: 5' GCTGCTCATCATCCCACTCTGG 3' |  |  |
| PLA2 | F: 5' GGCACAGAACAAGACGAT 3' | XB_001145489.2 | 126 |
|  | R: 5' CAGACGAACATCTCACACT 3' |  |  |
| GAPDH | F: 5' GCTGTGGAGAGATGGCAGAGGT 3' | NM_001004414 | 222 |
|  | R: 5' ACGGCAGGTCAGGTCAACAACA 3' |  |  |
| IL-10 | F: 5' AGCCAGCACTGCCACAAGAAC 3' | NM_204961.2 | 142 |
|  | R: 5' TAGTGTTGCCTGCTGCCGTG 3' |  |  |
| iNOS | F: 5' TGTGGAAGGACCGAGCTGTTGT 3' | NM_204305.2 | 163 |
|  | R: 5' CCTCCTCGCACACGGTACTCAT 3' |  |  |
| TNF-α | F: 5' CTCAGGACAGCCTATGCCAACA 3' | NM_204267.2 | 177 |
|  | R: 5' CCACCACACGACAGCCAAGT 3' |  |  |
| IL-1β | F: 5' AGCAGCAGCCTCAGCGAAGA 3' | NM_204524.2 | 183 |
|  | R: 5' CCTCCGCAGCAGTTTGGTCAT 3' |  |  |
| IL-2 | F: 5' CCGTGGCTAACTAACCTGCTGT 3' | NM_204153.2 | 110 |
|  | R: 5' CCCGTAGGGCTTACAGAAAGGA 3' |  |  |
| IL-6 | F: 5' AATCCCTCCTCGCCAATCT 3' | NM_204628.2 | 102 |
|  | R: 5' TCACGGTCTTCTCCATAAACG 3' |  |  |
| IFN-γ | F: 5' CACTGACAAGTCAAAGCCGC 3' | NM_205149.2 | 131 |
|  | R: 5' TCAAGTCGTTCATCGGGAGC 3' |  |  |
| IL-4 | F: 5' AGCCAGCACTGCCACAAGAAC 3' | NM_001007079.2 | 112 |
|  | R: 5' TAGTGTTGCCTGCTGCCGTG 3' |  |  |
| TGF-β1 | F: 5' GCCGACACGCAGTACACCAA 3' | NM_001318456.1 | 168 |
|  | R: 5' CTCCAGGTCCAGCCGTTG 3' |  |  |
