# Supplementary figures and images for: Effects of Salmonella Typhimurium infection on intestinal flora and intestinal tissue arachidonic acid metabolism in Wenchang chickens
Source: Front Microbiol. 2025 Jan 24;16:1514115. doi: 10.3389/fmicb.2025.1514115 (PMC11803450; doi:10.3389/fmicb.2025.1514115)

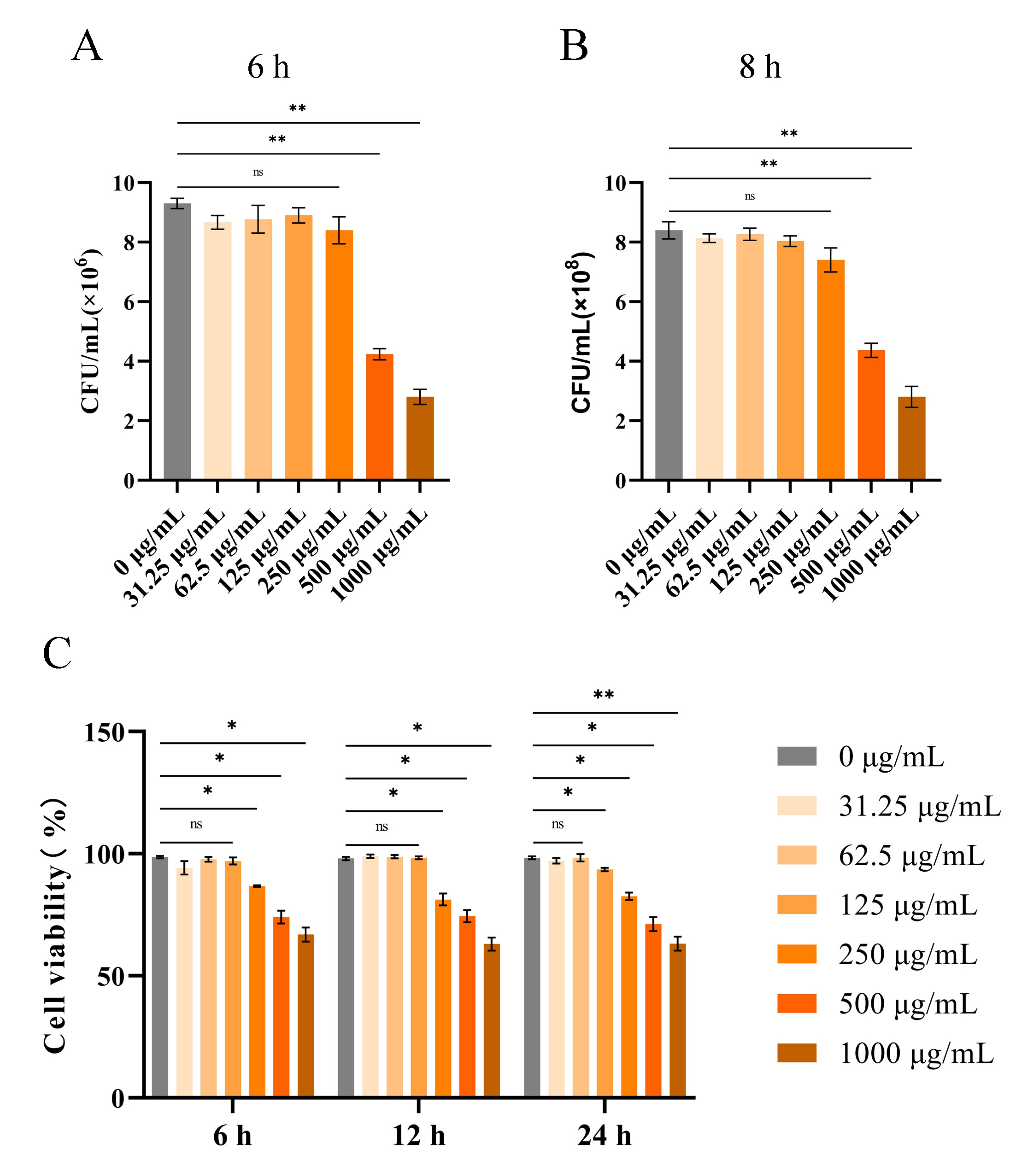

Supplement: Supplementary file 1 [file Data_Sheet_1.zip › Supplementary File(s)/Supplementary Figures/Figure S9.jpg]

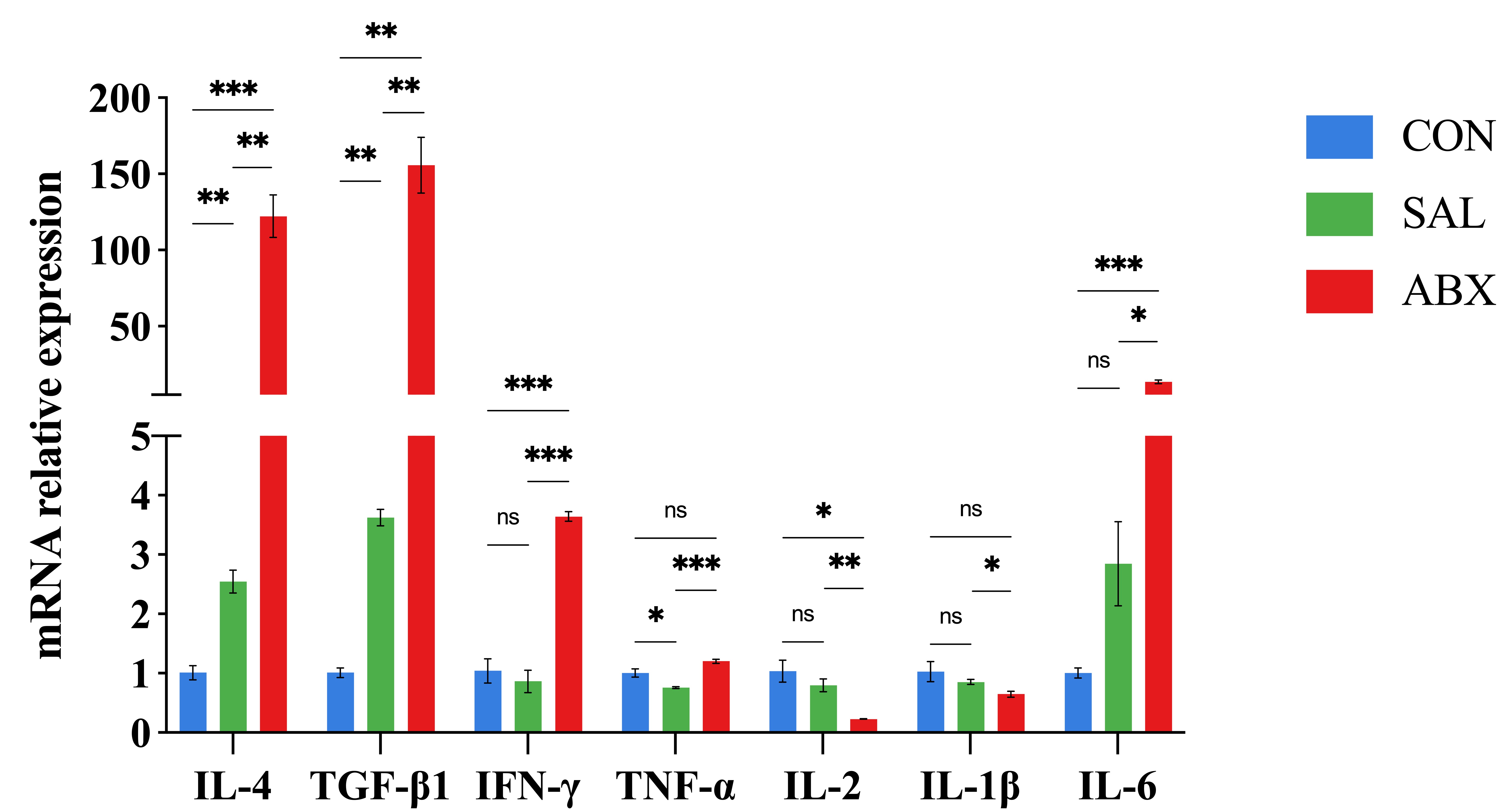

Supplement: Supplementary file 1 [file Data_Sheet_1.zip › Supplementary File(s)/Supplementary Figures/Figure S8.jpg]

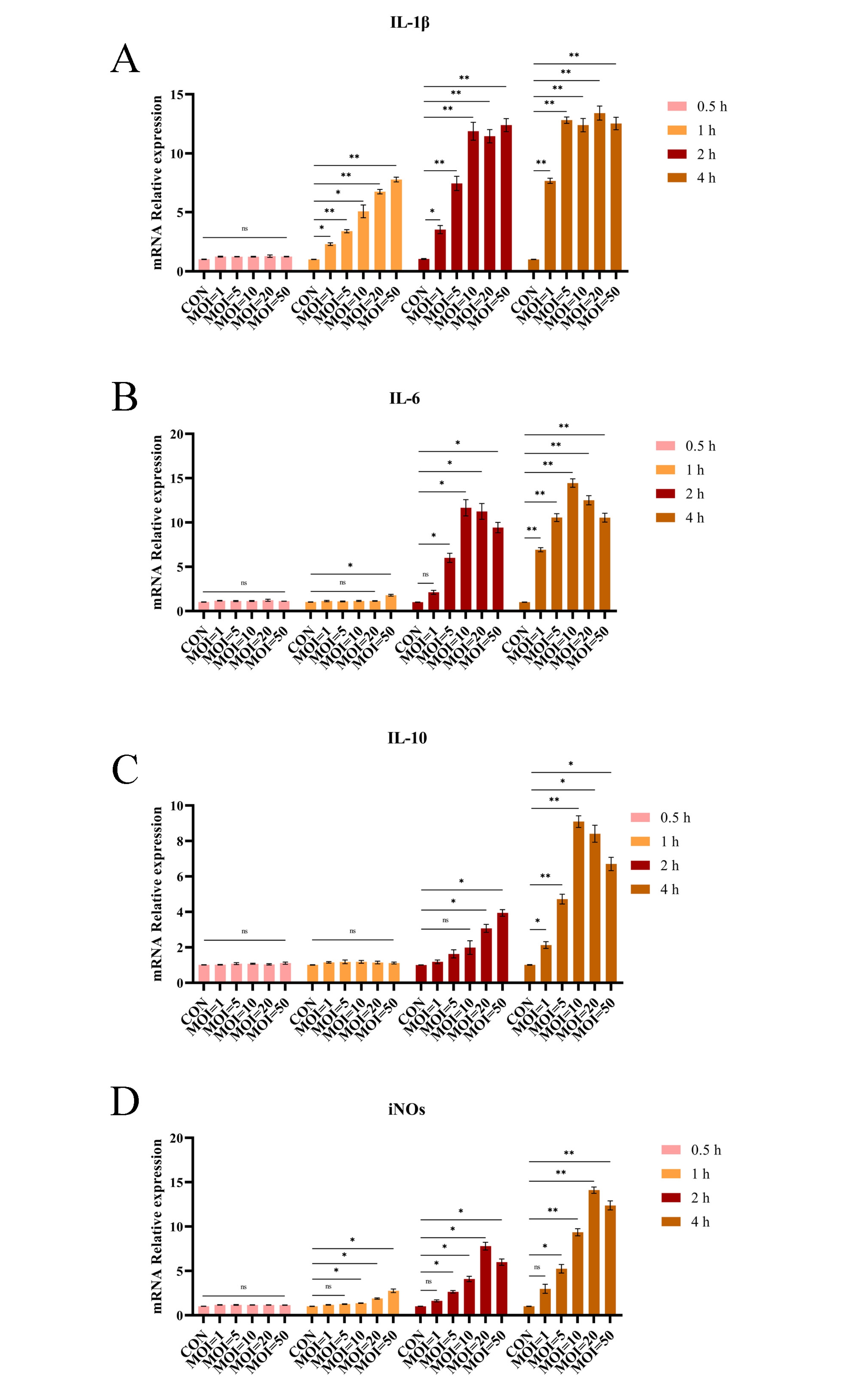

Supplement: Supplementary file 1 [file Data_Sheet_1.zip › Supplementary File(s)/Supplementary Figures/Figure S10.jpg]

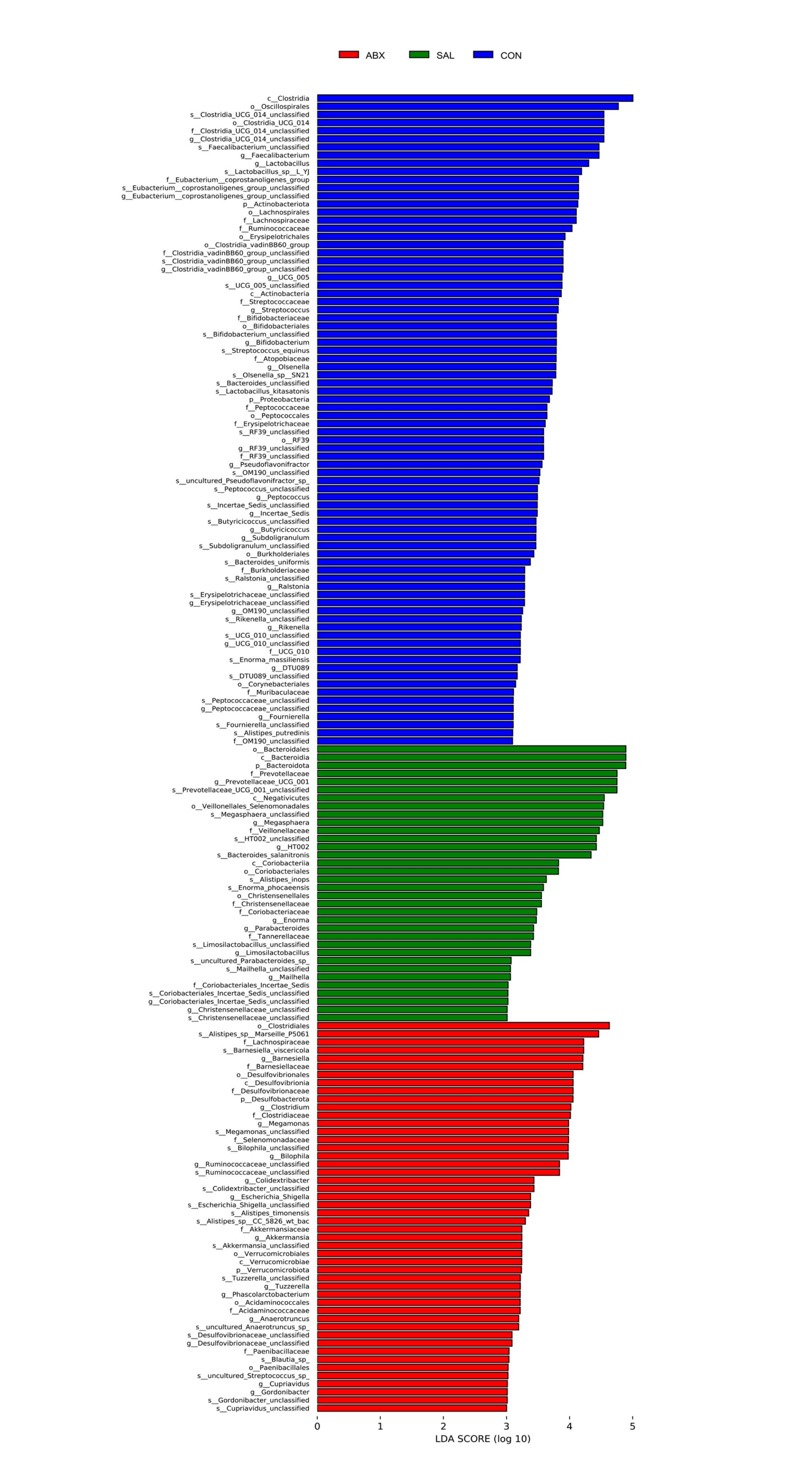

Supplement: Supplementary file 1 [file Data_Sheet_1.zip › Supplementary File(s)/Supplementary Figures/Figure S3.jpg]

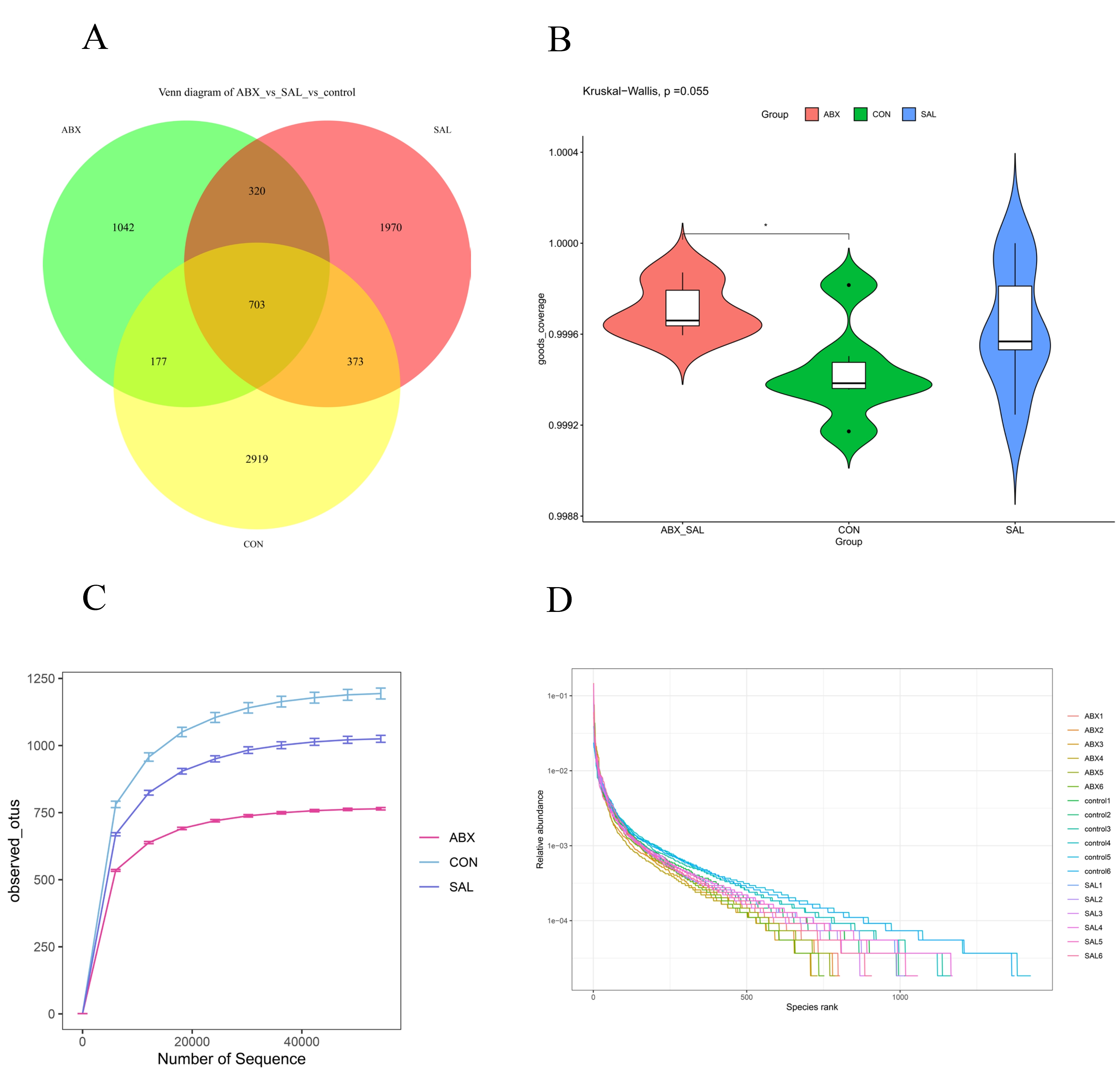

Supplement: Supplementary file 1 [file Data_Sheet_1.zip › Supplementary File(s)/Supplementary Figures/Figure S2.jpg]

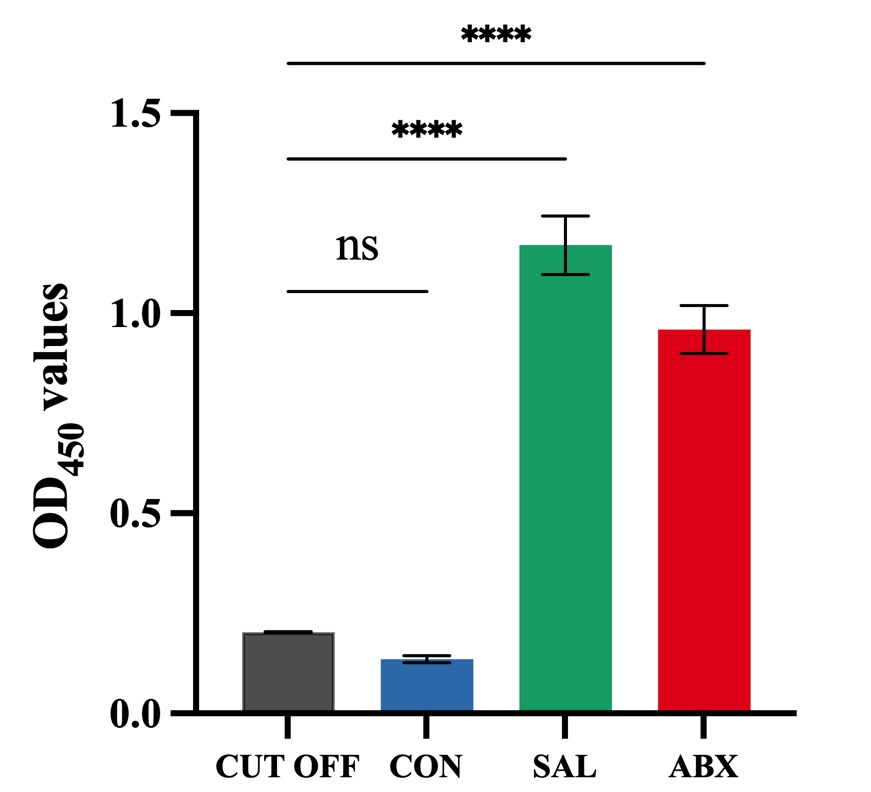

Supplement: Supplementary file 1 [file Data_Sheet_1.zip › Supplementary File(s)/Supplementary Figures/Figure S1.jpg]

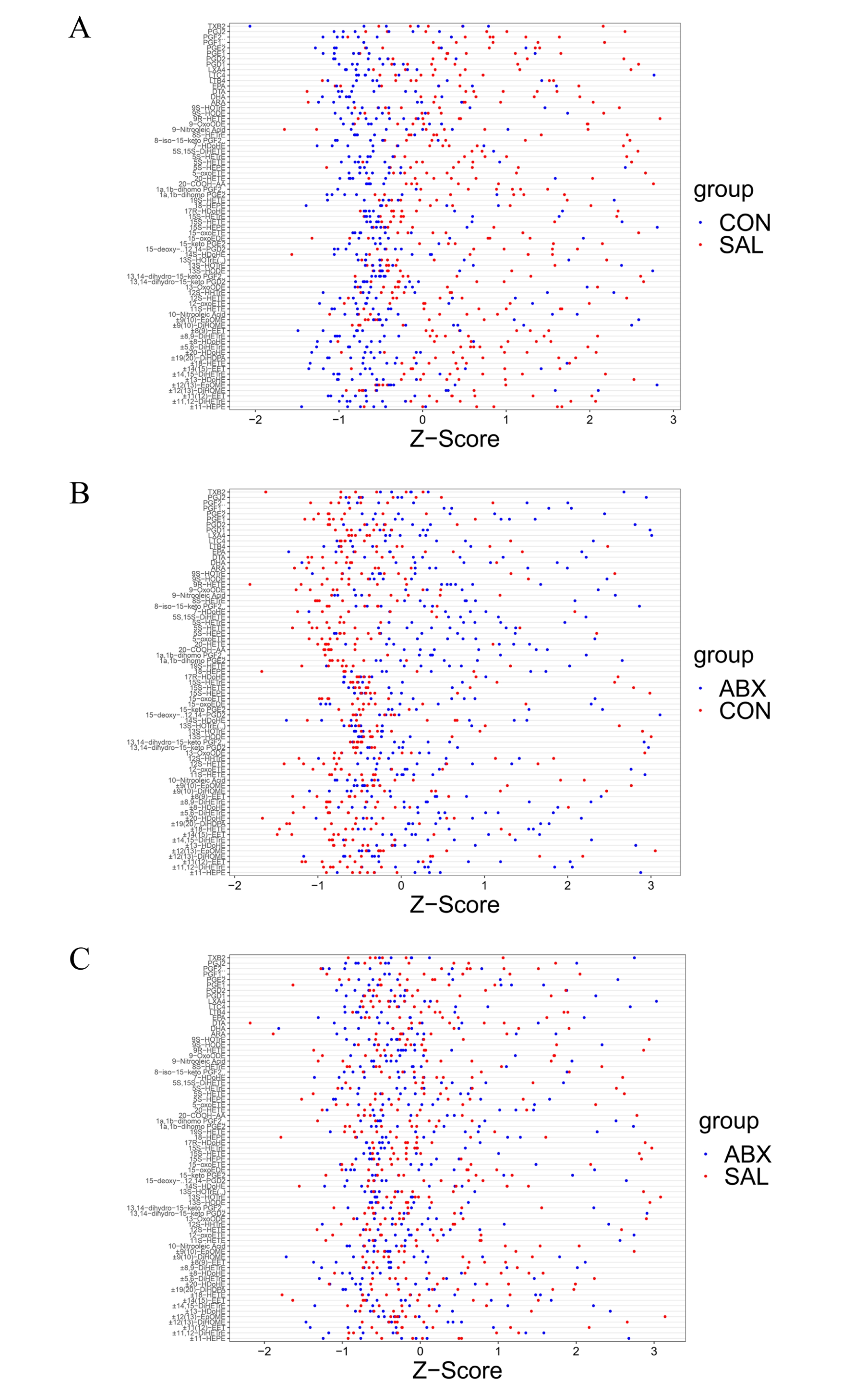

Supplement: Supplementary file 1 [file Data_Sheet_1.zip › Supplementary File(s)/Supplementary Figures/Figure S5.jpg]

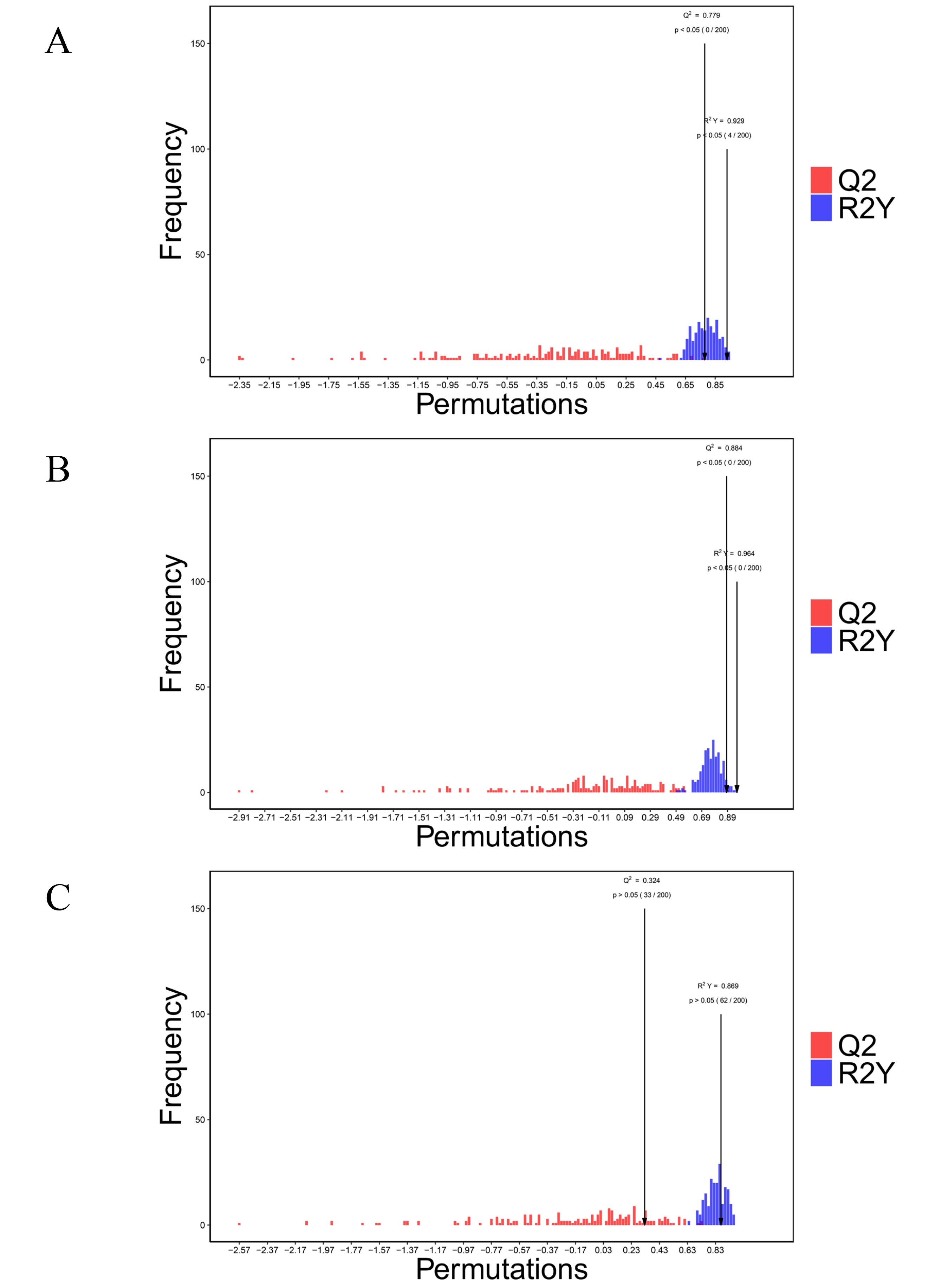

Supplement: Supplementary file 1 [file Data_Sheet_1.zip › Supplementary File(s)/Supplementary Figures/Figure S4.jpg]

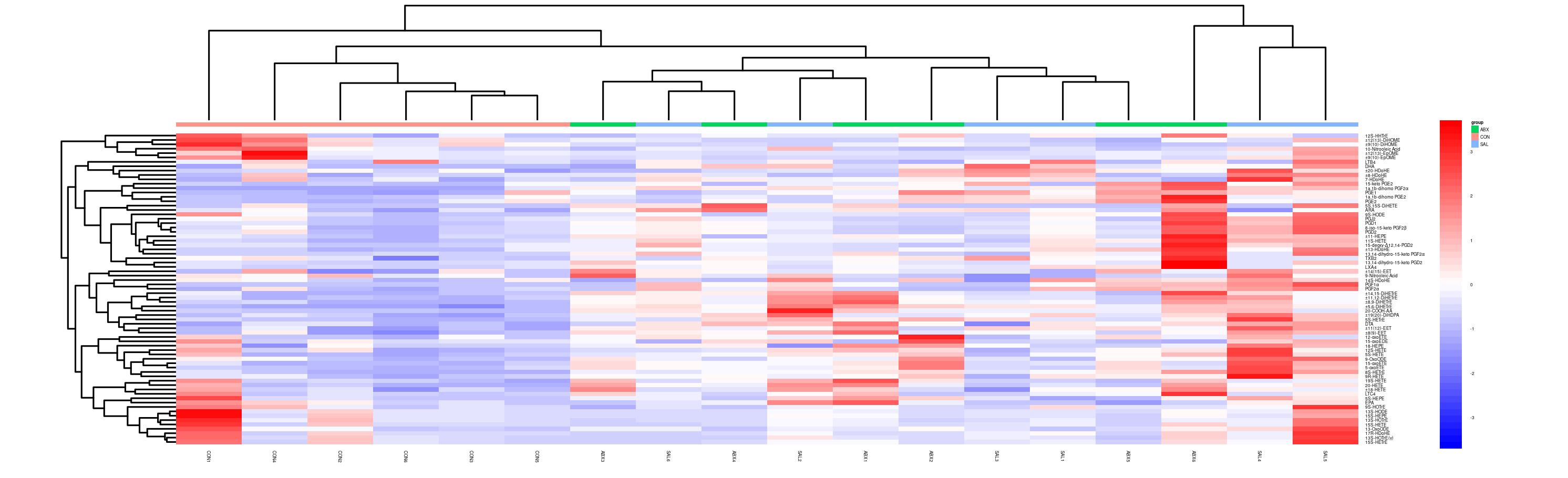

Supplement: Supplementary file 1 [file Data_Sheet_1.zip › Supplementary File(s)/Supplementary Figures/Figure S6.jpg]

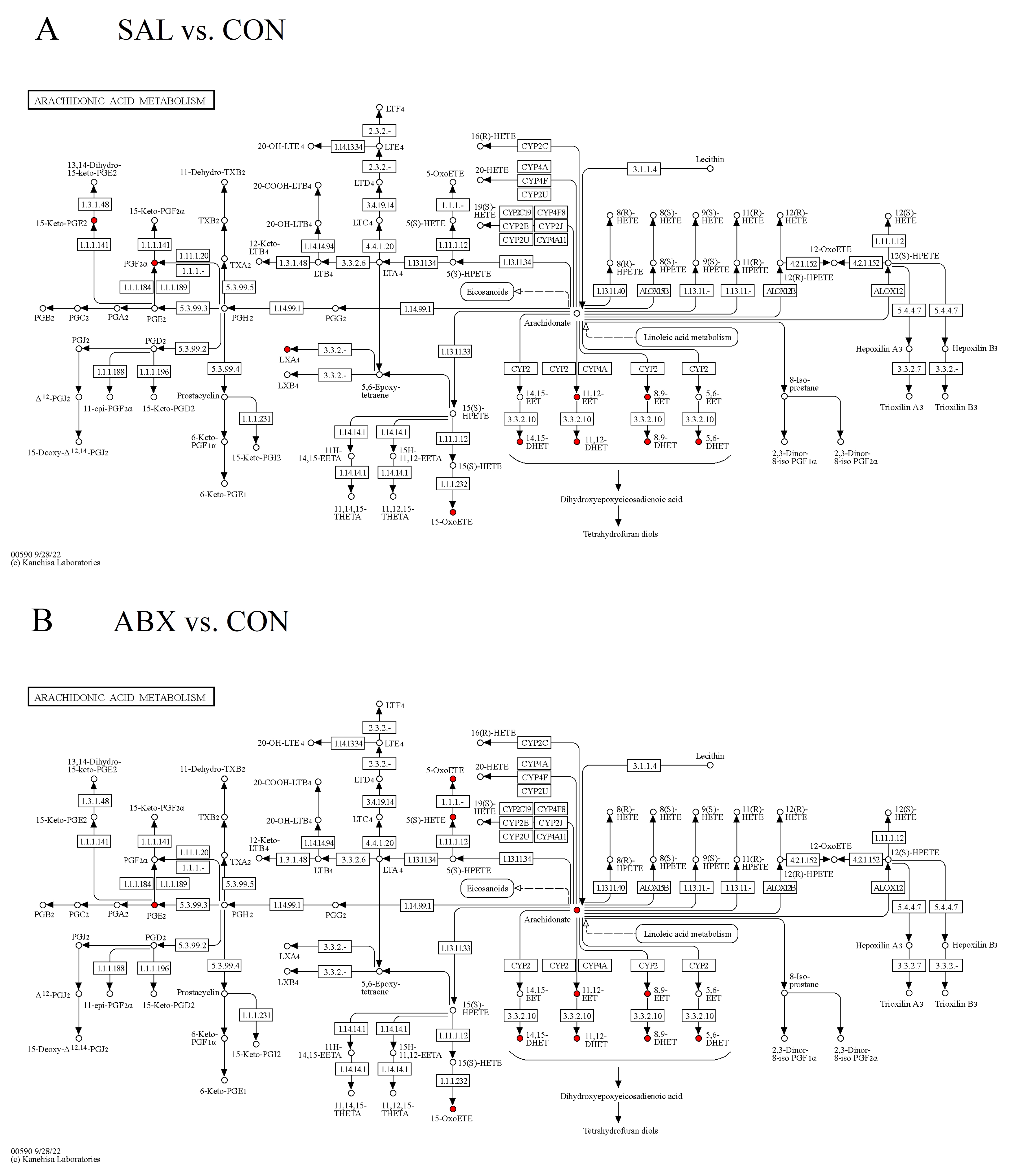

Supplement: Supplementary file 1 [file Data_Sheet_1.zip › Supplementary File(s)/Supplementary Figures/Figure S7.jpg]
